# Radiomodulatory effect of a non-electrophilic NQO1 inducer identified in a screen of new 6, 8-diiodoquinazolin-4(*3H*)-ones carrying a sulfonamide moiety

**DOI:** 10.1016/j.ejmech.2020.112467

**Published:** 2020-08-15

**Authors:** Aiten M. Soliman, Heba M. Karam, Mai H. Mekkawy, Maureen Higgins, Albena T. Dinkova-Kostova, Mostafa M. Ghorab

**Affiliations:** a Department of Drug Radiation Research, National Center for Radiation Research and Technology (NCRRT), Egyptian Atomic Energy Authority (EAEA), Nasr City P.O. Box 29, Cairo, 11765, Egypt; b Jacqui Wood Cancer Centre, Division of Cellular Medicine, School of Medicine, University of Dundee, Dundee, DD1 9SY, Scotland, UK; c Department of Pharmacology and Molecular Sciences and Department of Medicine, Johns Hopkins University School of Medicine, Baltimore, MD, 21205, USA

**Keywords:** Diiodoquinazolinone, Sulfonamide, Radiomodulatory, NQO1, Nrf2, Oxidative stress, Docking

## Abstract

Fifteen new quinazolinone derivatives bearing benzenesulfonamide moiety with variable acetamide tail were synthesized. The structures assigned to the products were concordant with the microanalytical and spectral data. Compounds **4–18** were screened for their ability to induce the antioxidant enzyme NAD(P)H: quinone oxidoreductase 1 (NQO1) in cells, a classical target for transcription factor nuclear factor erythroid 2-related factor 2 (Nrf2). The 2-((6,8-diiodo-4-oxo-3-(4-sulfamoylphenyl)-3,4-dihydroquinazolin-2-yl)thio)-*N*-(3,4,5-trimethoxyphenyl) acetamide **15** showed the most potent NQO1 inducer activity *in vitro*. Compound **15** had low toxicity in mice (LD_50_ = 500 mg/kg). It also reduced the damaging effects of gamma radiation, as assessed by the levels of Nrf2, NQO1, reactive oxygen species (ROS) and malondialdehyde (MDA) in liver tissues. In addition, compound **15** showed amelioration in the complete blood count of irradiated mice and enhanced survival over a period of 30 days following irradiation. Molecular docking of **15** inside the Nrf2-binding site of Kelch-like ECH associated protein 1 (Keap1), the main negative regulator of Nrf2, showed the same binding interactions as that of the co-crystallized ligand considering the binding possibilities and energy scores. These findings suggest that compound **15** could be considered as a promising antioxidant and radiomodulatory agent.

## Highlights

• Novel 6,8-diiodoquinazolinone derivatives carrying sulfonamide moiety were synthetized.
• Compounds **4-18** were assayed *in vitro* for their NQO1 inducer activity.
• Acute toxicity of the most potent NQO1 inducer **15** was low *in vivo* (LD_50_ = 500 mg/kg).
• Compound **15** inhibited oxidative stress and improved survival in irradiated mice.
• Molecular docking showed interactions of **15** with the Nrf2-binding site of Keap1.

## Graphical abstract

**Figure:**
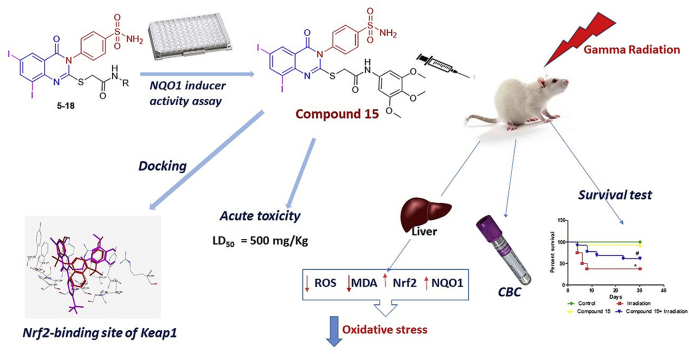

## 1 Introduction

The extensive use of radiotherapy and the damage caused to the surrounding normal organs have provoked researchers to find new strategies to protect normal tissues from radiation hazards [1,2]. The risk of injury from radiation can diminish the value of radiotherapy and contribute to complications for long-term cancer survivors [3]. Ionizing radiation interrupts cell functions through radiolysis of water and the production of reactive oxygen species (ROS) or reactive nitrogen species (RNS) [4,5]. Excessive production of ROS and RNS promotes oxidative stress, which can affect all cellular components, including single or double DNA strand breaks [6]. This ROS-mediated toxicity can lead to mutations and consequently cause cardiovascular, neurological toxicities and sexual dysfunction as well as cancer [[7], [8], [9], [10]]. In order to reduce these radiation-induced side effects, radioprotective drugs are used [11]. Also, the use of multi-target antioxidants that act as radioprotectors can help limit normal tissue damage caused by ionizing radiation [[12], [13], [14]].

Nuclear factor erythroid 2-related factor 2 (Nrf2) is a transcription factor that regulates the expression of various antioxidant proteins to protect against oxidative damage in the cell [15]. The abundance of Nrf2 is negatively regulated by Kelch-like ECH associated protein 1 (Keap1), a substrate adaptor for a Cullin3/Rbx1 ubiquitin ligase that binds and continuously targets Nrf2 for ubiquitination and proteasomal degradation [[16], [17], [18]]. Under conditions of oxidative stress, redox-sensitive cysteine sensors of Keap1 are modified leading to loss of, its ability to target Nrf2 for degradation; consequently, Nrf2 transports into the nucleus where it initiates the transcription of its downstream target genes, such as NAD(P)H: quinone oxidoreductase1 (NQO1) [19].

Quinazolinone is a strategic scaffold that has a wide range of pharmacological activities such as antioxidant, anti-inflammatory and anticancer activities [[20], [21], [22], [23]]. Sulfonamides, in addition to their use as antibiotics [[24], [25], [26], [27]], have many pharmacological activities and can be used as antiviral [28], anti-inflammatory [29], antioxidant [30,31], and anticancer agents [[32], [33], [34], [35]]. These versatile pharmacological activities make the two chemical classes excellent candidates for developing new multi-target agents through a slight alteration in the structure that might lead to diversity in the biological activity [20,36,37]. In addition, numerous studies have revealed iodine to be a potent antioxidant with higher potency than that of ascorbic acid [38,39]. Iodine can act as an electron donor that quenches ROS, such as OH^•^ and H_2_O_2_ [40], or decreases the damaging effects of ROS, thus increasing the total antioxidant status in human serum [41].

In this context, it seemed of interest to search for new compounds with the ability to scavenge ROS and protect cells. A series of new 6,8-diiodoquinazolin-4(*3H*)-one conjugated to benzenesulfonamide was synthesized by the introduction of the sulfonamide group at the N-3 of quinazolinone with the incorporation of varying acetamide terminal aimed at exploring the potential antioxidant and radioprotective activity. The antioxidant potential of the target compounds was first measured using a quantitative and robust NQO1 inducer activity bioassay in cells. Acute toxicity study for the most active compound was then performed *in vivo*. A non-toxic dose was subsequently selected to investigate the potential protective effect against whole-body gamma irradiation-induced oxidative stress in experimental mice. All groups were observed 30 days after irradiation for survival and weight changes. Additionally, molecular docking was performed inside the Nrf2-binding site of Keap1 to gain insights into the molecular interactions and possible mode of action.

## 2 Results and discussion

### 2.1 Chemistry

**Scheme 1:**
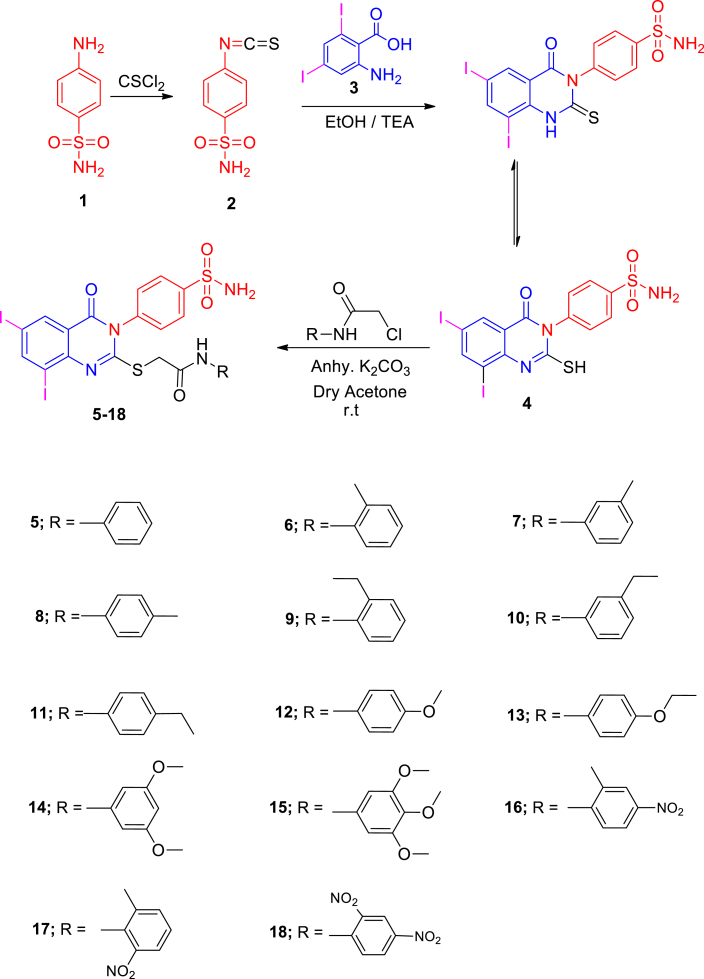
The synthetic pathways for the development of the diiodoquinazolinone derivatives **4–18.**

Scheme 1 shows the synthesis of thioacetamide quinazolinone benzenesulfonamide derivatives **5–18**. The starting material 4-(6,8-diiodo-2-mercapto-4- oxoquinazolin-3(4*H*)-yl) benzenesulfonamide **4** was prepared from the reaction of 4-isothiocyanatobenzenesulfonamide **2** [42] and 2-amino-3,5-diiodobenzoic acid **3**. The coupling of **4** with the 2-chloro-*N*-substituted acetamide in dry acetone and anhydrous K_2_CO_3_ yielded the corresponding 2-((6,8-diiodo-4-oxo-3-(4-sulfamoylphenyl)-3,4-dihydroquinazolin-2-yl)thio)-*N*-substituted acetamide **5–18**. IR spectra of **5**–**18** displayed additional NH, CH_2_ aliphatic and CO bands at their specified regions. ^1^H NMR spectra of **5**–**18** revealed the acetamide group through the presence of two singlets, one at 4.17–4.31 ppm referring to the CH_2_ and the other at 9.66–11.21 ppm attributed to the NH protons with the disappearance of SH singlet of **4** at 1.97 ppm. ^13^C NMR of **5**–**18** exhibited two signals peculiar to the CH_2_ and CO carbons. ^1^H NMR spectra of **6**–**8** displayed singlets at 2.21, 2.28 and 2.30 ppm assigned to the CH_3_ group at the *ortho*, *meta* and *para-*positions of the phenyl group. ^13^C NMR of **6**–**8** showed signals at 16.32, 24.13 and 19.21 ppm for the CH_3_ group. ^1^H NMR spectra of **9**–**11** revealed triplets at 1.31, 1.20 and 1.15 ppm attributed to the CH_3_ ethyl, and quartet at 2.54, 2.58 and 2.55 ppm referring to the CH_2_ ethyl at the *ortho*, *meta* and *para-*positions. ^13^C NMR of **9**–**11** showed two signals at 14.67, 15.21, 17.12 due to CH_3_ ethyl and 24.23, 24.10, 29.40 due to the CH_2_ ethyl groups, respectively. ^1^H NMR spectra of **12** revealed singlet at 3.75 ppm attributed to the OCH_3_ protons, while ^13^C NMR of **12** showed a signal at 54.26 ppm due to the OCH_3_ carbon. ^1^H NMR spectra of **13** revealed triplet at 1.27 ppm and quartet at 3.97 ppm due to the ethoxy group. ^1^H NMR spectra of **14** revealed a singlet at 3.74 ppm due to the 2OCH_3_ protons, while **15** revealed two singlets at 3.70 and 3.81 ppm due to the 3OCH_3_ protons. IR of **16**–**18** showed NO_2_ bands.

### 2.2 Biological activity

#### 2.2.1 In vitro screening

**Fig. 1:**
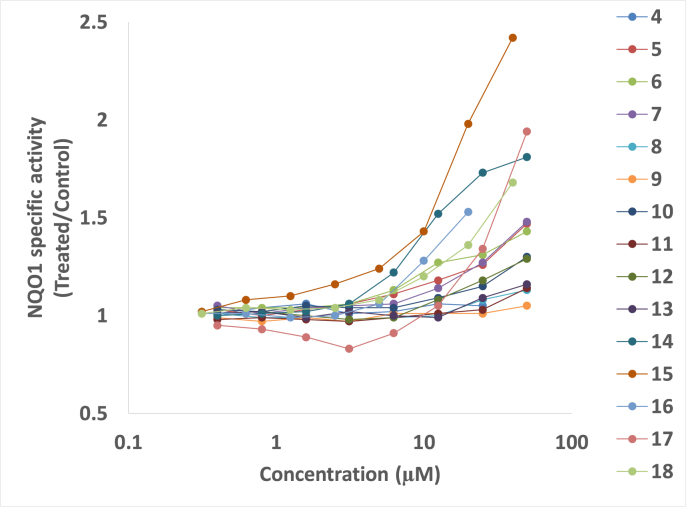
Concentration dependence of the NQO1 inducer activity of compounds **4–18**.

The antioxidant activity of compounds **4–18** was screened using the NQO1 inducer activity assay. The Concentration of the novel compounds to Double the specific enzyme activity of NQO1 (CD value) was used as a measure of inducer potency and results obtained are presented in Fig. 1 & Table 1. Evaluation of the NQO1 inducer activity showed that compounds **4, 8, 9, 11** and **13** were inactive, whereas compounds **5**, **6**, **7, 10, 12, 14** and **18** had activity; however, CD value was not reached. Compounds **15** (CD = 20 μM), and **17** (CD = 50 μM) showed concentration-dependent inducer activity. These diiodoquinazolinones represent a new chemical class of NQO1 inducers, thus adding to the existing knowledge of the diversity of the many chemical scaffolds that have been reported to induce this antioxidant enzyme. The classical NQO1 inducers are primarily oxidants and electrophiles or other compounds that react (or are metabolized to products that react) and chemically modify cysteine sensors of Keap1 [43]. A new generation of NQO1 inducers is also emerging, that of noncovalent small-molecule modulators of the Keap1–Nrf2 protein-protein interaction [[44], [45], [46]]. Because our diiodoquinazolinones have some common features with the Keap1–Nrf2 protein-protein interaction inhibitors, in this study we tested the potential ability of these compounds to directly disrupt the binding of Keap1 to Nrf2 by molecular modeling (see section 2.3).

**Table 1:** NQO1 inducer activity and CD values of compounds **4**–**18**.

Conc. (μM)	Compound no.														
	4	5	6	7	8	9	10	11	12	13	14	15	16	17	18
**0.313**	NR	NR	NR	NR	NR	0.99	1.03	NR	NR	1.01	NR	1.02	1.01	NR	1.01
**0.4**	1.01	1.03	1.04	1.05	1.01	NR	NR	0.98	1.01	NR	1.00	NR	NR	0.95	NR
**0.625**	NR	NR	NR	NR	NR	0.97	1.02	NR	NR	1.02	NR	1.08	1.01	NR	1.04
**0.8**	1.04	1.00	1.04	1.01	0.99	NR	NR	0.99	1.02	NR	1.01	NR	NR	0.93	NR
**1.25**	NR	NR	NR	NR	NR	0.99	1.05	NR	NR	0.99	NR	1.10	0.99	NR	1.03
**1.6**	1.06	1.03	1.04	1.04	0.99	NR	NR	0.98	1.00	NR	1.02	NR	NR	0.89	NR
**2.5**	NR	NR	NR	NR	NR	0.97	1.04	NR	NR	1.02	NR	1.16	1.00	NR	1.04
**3.125**	1.01	1.06	1.06	1.05	0.97	NR	NR	0.97	0.98	NR	1.06	NR	NR	0.83	NR
**5**	NR	NR	NR	NR	NR	1.01	1.04	NR	NR	1.00	NR	1.24	1.06	NR	1.08
**6.25**	1.02	1.11	1.13	1.06	0.99	NR	NR	0.99	0.99	NR	1.22	NR	NR	0.91	NR
**10**	NR	NR	NR	NR	NR	1.01	1.09	NR	NR	0.99	NR	1.43	1.28	NR	1.20
**12.5**	1.06	1.18	1.27	1.14	1.00	NR	NR	1.01	1.08	NR	1.52	NR	NR	1.05	NR
**20**	NR	NR	NR	NR	NR	1.01	1.15	NR	NR	1.09	NR	1.98	1.53	NR	1.36
**25**	1.05	1.26	1.31	1.27	1.08	NR	NR	1.03	1.18	NR	1.73	NR	NR	1.34	NR
**40**	NR	NR	NR	NR	NR	1.05	1.30	NR	NR	1.16	NR	2.42	NR	NR	1.68
**50**	NR	1.47	1.43	1.48	1.13	NR	NR	1.14	1.29	NR	1.81	NR	NR	1.94	NR
**CD**^a^	NR	NR	NR	NR	NR	NR	NR	NR	NR	NR	NR	**20**	NR	**50**	NR

NR means not recorded.

a CD values are the averages of three independent experiments, each with eight replicate wells of cells, and SD for each data point was within 5% of the value.

#### 2.2.2 In vivo evaluation

##### 2.2.2.1 Determination of toxicity (lethal dose fifty, LD_50_) of compound **15**

The most promising compound, **15,** was investigated *in vivo* for acute toxicity (LD_50_) in albino mice, and the value was found to be 500 mg/kg body weight (i.p.). Subsequently, one-tenth of this dose was selected as the therapeutic dose for further evaluation of the potential radioprotective effects of compound **15**.

##### 2.2.2.2 Evaluation of the radiomodulatory effect of compound **15** in mice

Four groups of mice were used, the first group served as control, the second group was irradiated at a dose of 7 Gy as a single dose, the third group was injected i.p. with compound **15** only for 5 consecutive days and the last group received compound **15** then exposed to 7 Gy of gamma radiation. After 3 days from irradiation, five mice were checked for liver and hematopoietic system toxicities. The residual mice in all groups were monitored over 30 days to evaluate the survival rate and body weight changes.

###### 2.2.2.2.1 The effect of compound **15** on radiation-induced liver toxicity

Gamma radiation-induced hepatic oxidative stress as shown by a significant increase in hepatic levels of nuclear Nrf2 (1.3-fold), NQO1 (3.2-fold), ROS (1.5-fold) and the lipid peroxidation product malondialdehyde (MDA) (2-fold) as compared to non-irradiated (control) mice. This was in agreement with other studies [2,47].

Ionizing radiation is believed to induce damage through the generation of ROS, resulting in an imbalance in the oxidant/antioxidant ratio in cells [8,48]. In the current experiment, the presence of ROS-mediated damage was confirmed by the increase in MDA levels in irradiated liver, in addition to the increase in the expression of the enzymatic antioxidant system. Moreover, these results support the notion that Nrf2 is an initial regulator of cellular responses to radiation exposure [49]. Once Nrf2 translocates to the nucleus it induces expression of endogenous antioxidant enzymes, such as NQO1 [50], a flavoprotein involved in cellular protection against oxidative stress [51].

**Fig. 2:**
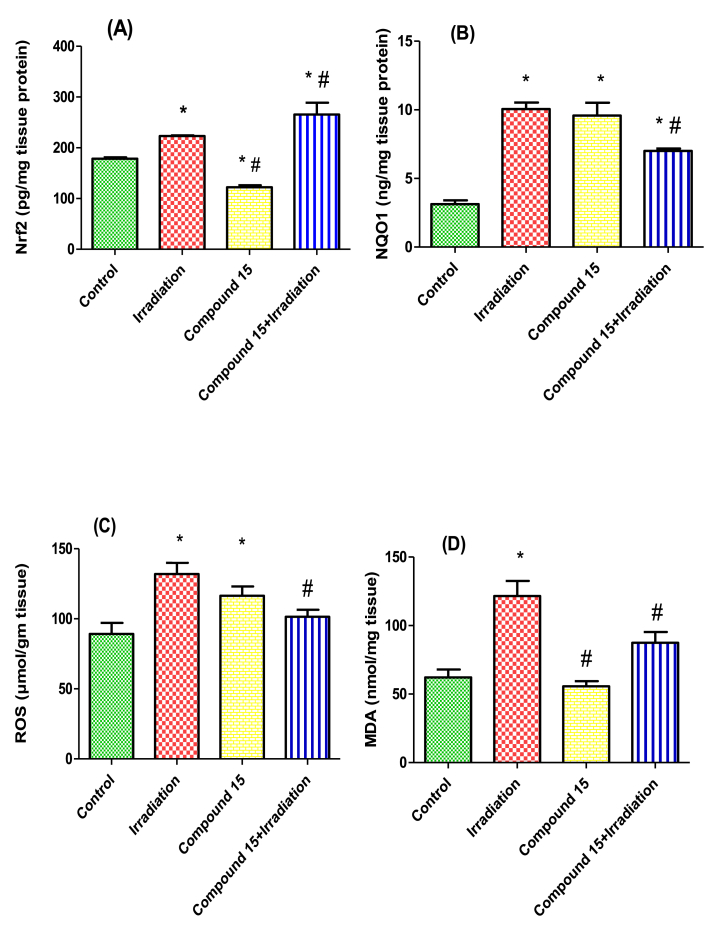
Effect of compound **15** on (A) Nrf2, (B) NQO1, (C) ROS and (D) MDA levels in liver of non-irradiated (control) and irradiated mice after 3 days of irradiation. The results were expressed as mean ± S.E. Statistical analysis was carried out by one-way ANOVA followed by Bonferroni’s multiple comparison test. ∗: significantly different from control group, #: significantly different from irradiated group at *p* < 0.05. (n = 5).

**Fig. 3:**
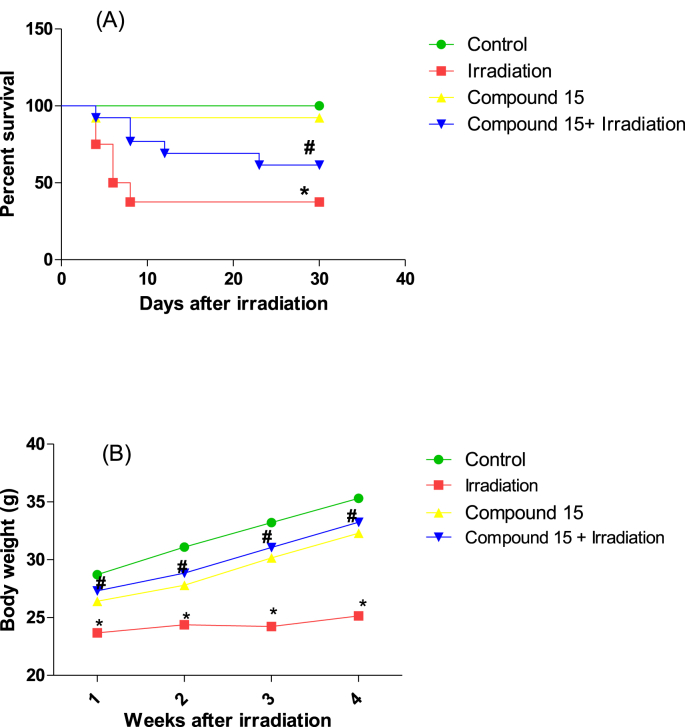
A) Survival percent and B) Body weight changes of control, irradiated, compound **15** and compound **15** + irradiated mice through 30 days after irradiation. The results were expressed as mean ± S.E. (n = 15). Statistical analysis was carried out by Kaplan-Meier method followed by the Mantel–Cox test for survival analysis. Body weight changes between groups were analyzed by two-way ANOVA followed by Bonferroni’s post test. ∗: significantly different from control group, #: significantly different from irradiated group at *p* < 0.05.

**Fig. 4:**
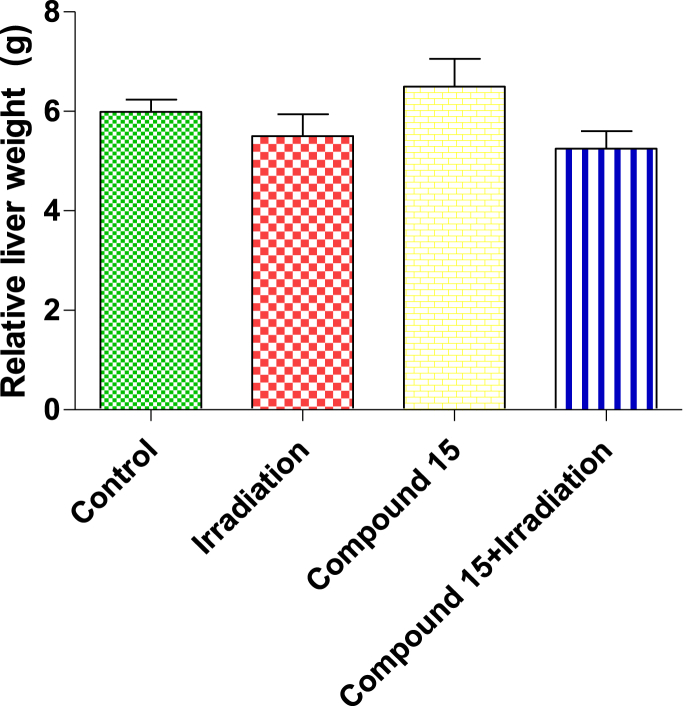
Effect of compound **15** on relative liver weight in non-irradiated (control) and irradiated mice after 3 days of irradiation. The results were expressed as mean ± S.E. (n = 5). Statistical analysis was carried out by one-way ANOVA followed by Bonferroni’s multiple comparison test. There were no significant differences between groups.

Treatment of non-irradiated mice with compound **15** led to an increase in NQO1 and ROS levels and a decrease in Nrf2, with no significant change in MDA level as compared to normal (non-irradiated) mice (Fig. 2). A significant increase in Nrf2 levels (19%) as well decrease in the levels of NQO1 (30%), ROS (23%) and MDA (28%) was observed in irradiated mice livers treated with compound **15** when compared to the group subjected to radiation alone (Fig. 2). Moreover, treatment with compound **15** improved both survival and body weight of the animals following irradiation (Fig. 3) without affecting the liver weight (Fig. 4) as compared to irradiated mice. The present results indicate that compound **15** has an antioxidant capacity as the treatment of irradiated mice with **15** prevents oxidative stress, reducing the increase in lipid peroxidation markers and maintaining the expression of Nrf2 compared with the irradiated group suggesting improved hepatic antioxidant capacity. Hence, compound **15** validated its radiomodulatory and antioxidant effect through its main structure; quinazolinone and sulfonamide that goes in line with Soliman *et al.* [52]. Also, this finding was reinforced by Cuadrado and his colleagues, who emphasized the importance of therapeutic targeting for Nrf2 because of its resourceful cytoprotective roles against a plethora of diseases that are associated with oxidative stress [53].

At the same time, it was found that NQO1 expression levels of irradiated mice treated with **15** were significantly lower as compared to vehicle-treated irradiated ones, but still significantly higher than normal levels. Interestingly, the levels of NQO1 in all experimental groups correlate with the levels of ROS, suggesting ROS involvement in the NQO1 induction. The lower levels of NQO1 and ROS in the irradiated group that also received **15** could be the results of increased antioxidant capacity due to Nrf2 activation [54]. Additionally, it has been reported that Nrf2 modifies ROS production partly by regulating NQO1 expression [55]. On the other hand, the NQO1 levels were significantly higher than the non-irradiated controls, in agreement with the cell culture results (this study). Notably, the increased levels of ROS in non-irradiated mice treated with compound **15** are consistent with the increased levels of ROS following genetic Nrf2 activation by Keap1 knockdown [54]. Importantly however, the increased ROS production that accompanies NQO1 induction does not lead to damage, as evidenced by the lack of increase in the levels of MDA (this study).

###### 2.2.2.2.2 The effect of compound **15** on the hematopoietic system

**Fig. 5:**
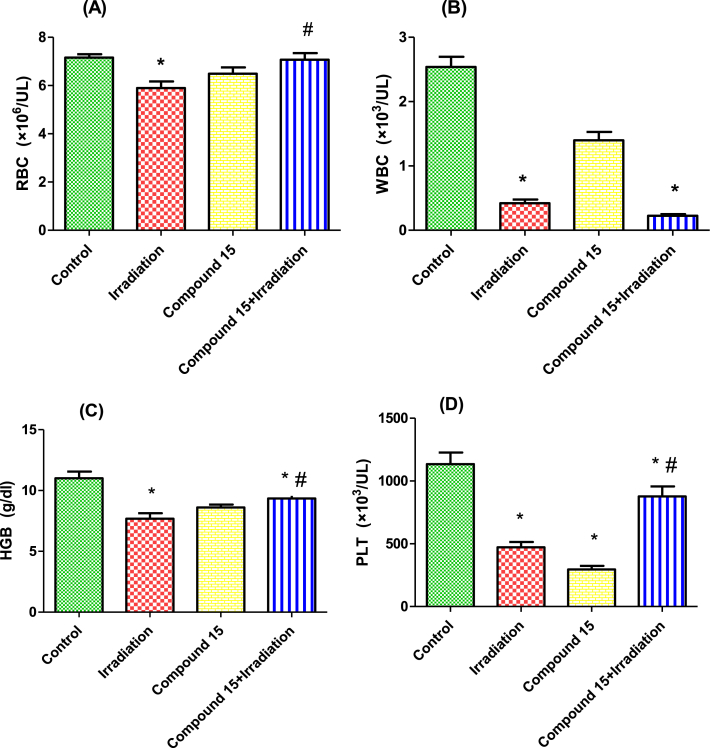
Effect of compound **15** on: (A) RBCs, (B) WBCs, (C) HGB concentration and (D) PLT counts in non-irradiated (control) and irradiated mice after 3 days of irradiation. The results were expressed as mean ± S.E. (n = 5). Statistical analysis was carried out by one-way ANOVA followed by Bonferroni’s multiple comparison test. ∗: significantly different from control group, #: significantly different from irradiated group.

To examine the possible role of compound **15** in protecting the hematopoietic system against irradiation, we measured the peripheral blood cell counts of red blood cells (RBCs), white blood cells (WBCs), hemoglobin (HGB) and platelets (PLT). The irradiated mice exhibited a significant decrease in RBCs, WBCs, HGB and PLT compared with the control group (Fig. 5). These results are mainly attributed to the fact that irradiation causes the formation of free radicals which initiate a chain of events leading to the decline in the levels of hematological parameters [56]. Indeed, it has been well established that gamma irradiation induces RBC injury, including morphological and quantitative changes of RBCs. These alternations may be partly attributed to radiation-induced oxidative stress in RBCs. Exposure to radiation results in the formation of reactive oxygen species (ROS) and reactive nitrogen species (RNS), as well as DNA damage, which can then lead to severe injury to the hematopoietic system [57]. This is in harmony with Wang *et al.* [58] who stated that, injury to the hematopoietic system is the most common injury induced by irradiation. This was attributed to the effect of ionizing radiation on hematopoietic stem cells and hematopoietic progenitor cells, which are principally responsible for hematopoietic recovery. Treatment of irradiated mice with compound **15** ameliorated the decrease in peripheral blood cells, particularly RBCs, HGB and PLT. Hence, the antioxidant properties of compound **15** may contribute to the amelioration of RBC counts and HGB in irradiated mice. This is consistent with other studies for antioxidants effects on the hematopoietic system [57,59]. This might be explained through the promotion effect of radioprotectors to proliferate hematopoietic stem cells and they also could increase the levels of leukocyte growth factors [60,61]. Besides, several potent radioprotectors protect various membrane systems, as well as hematopoietic stem cells from peroxidative damages, that happened after irradiation so it could protect blood components against irradiation [62]. Taken all together, these results demonstrate the protective effect of compound **15** against gamma radiation.

### 2.3 Molecular docking

Molecular docking was performed to assess the ability of compound **15** to block the Kelch domain of Keap1. Through its Kelch domain, Keap1 binds to Nrf2, promoting its degradation, resulting in low cytoprotective gene levels [63]. The PDB file: 4IQK was obtained from the Protein Data Bank. The binding site of Kelch domain has been reported to have five subpockets: P1, P2, P3, P4 and P5 [64]. P1 and P2 are positively charged pockets that contain the arginine triad (Arg 415, Arg 483 and Arg 380). This triad is crucial for the selectivity of the molecular recognition, together with a group of hydrophobic residues contributes to the stability of the complex. P1 is formed by residues Arg 415, Ile 461, Gly 462, Phe 478, Arg 483 and Ser 508. P2 is formed by Ser 363, Arg 380, Asn 382 and Asn 414. P3 is a neutrally charged pocket composed of Gly 509, Ser 555, Ala 556, Gly 571, Ser 602 and Gly 603. P4 is formed by Tyr 525, Gln 530 and Tyr 572, whereas P5 is formed by Tyr 334 and Phe 577.

**Fig. 6:**
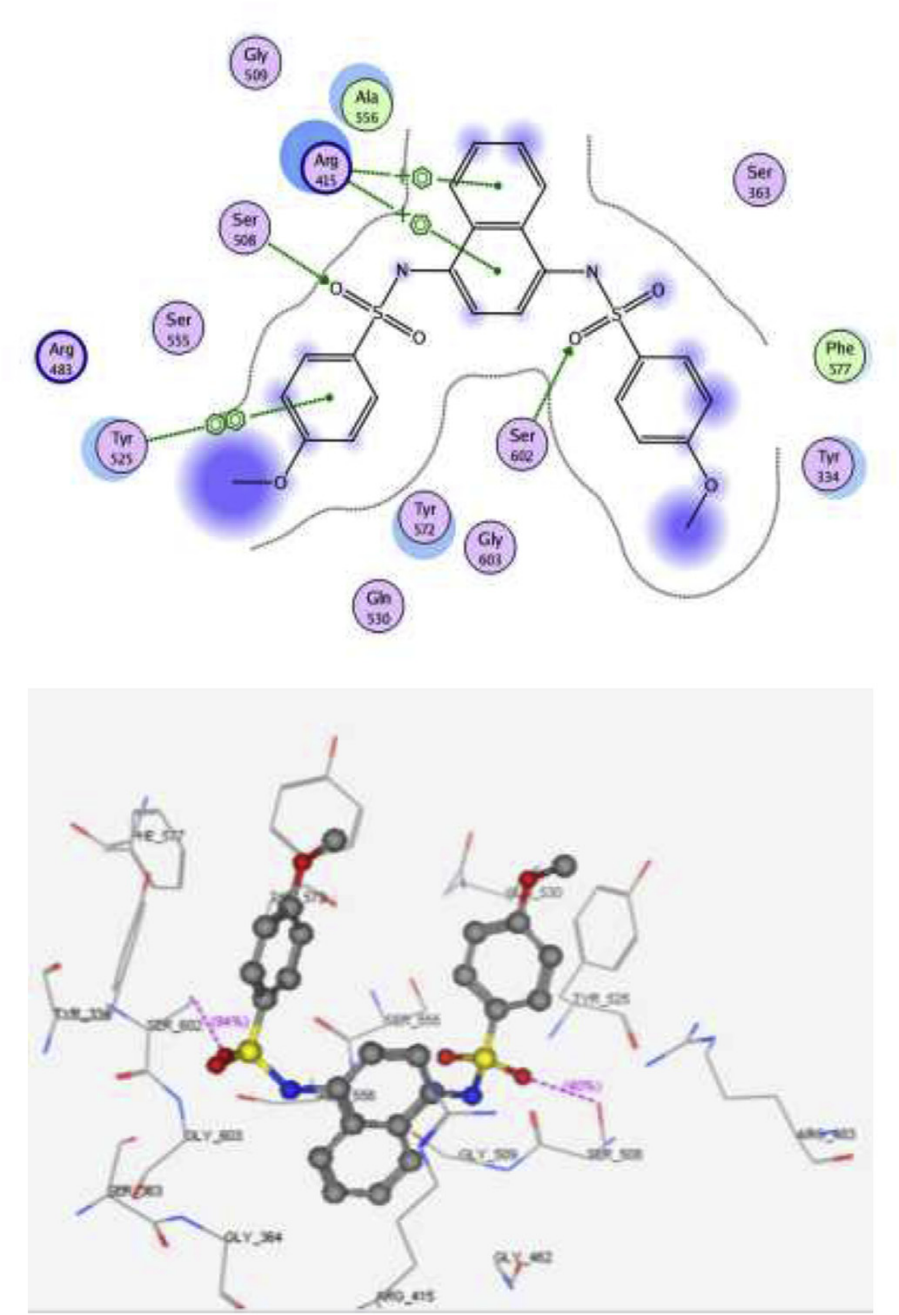
2D and 3D interaction poses of the *N,N′*-naphthalene-1,4-diylbis(4-methoxybenzenesulfonamide showing cation-π, π-π interaction and hydrogen bonds with the key amino acids inside the binding pocket.

**Fig. 7:**
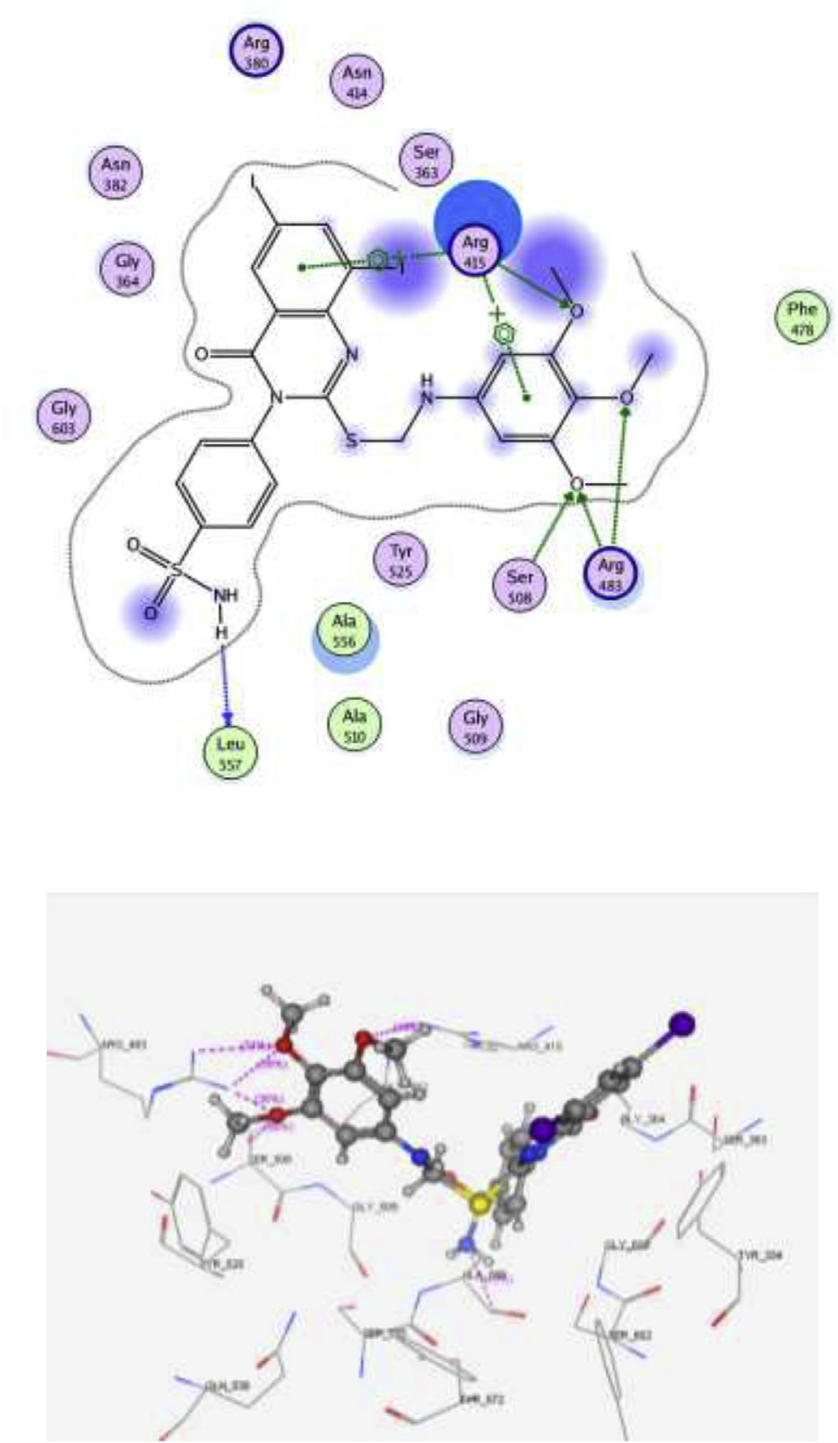
2D and 3D interaction pose of compound **15** showing cation-π, π-π interactions inside the binding pocket of 4IQK.

**Fig. 8:**
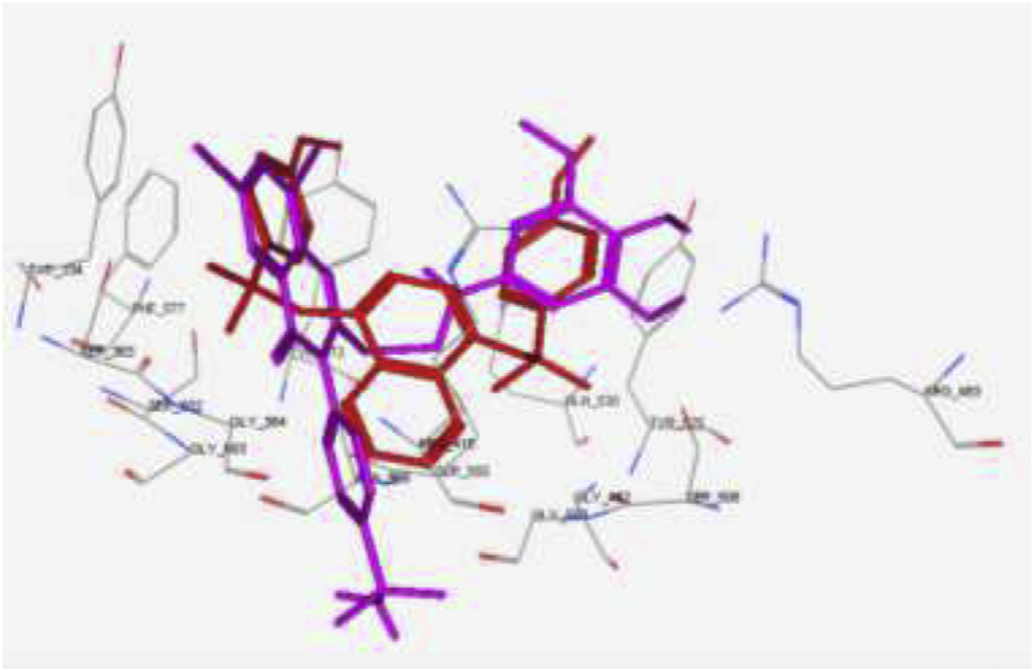
Superimposition of compound **15** (magenta) and the co-crystallized ligand (red) showed that they adopt the same orientation inside the receptor. (For interpretation of the references to color in this figure legend, the reader is referred to the Web version of this article.)

The main interactions observed by the co-crystallized ligand (*N, N′*-naphthalene-1,4-diylbis(4-methoxybenzenesulfonamide) are two cation-pi interaction with Arg 415, pi–pi interaction with Tyr 525 and two hydrogen bonds with Ser 602 and Ser 508, with S = -10.11 kcal/mol (Fig. 6). Compound **15** showed the same key interactions exhibited by the co-crystallized ligand. Compound **15** (S = -9.61 kcal/mol, RMSD = 1.34 Å) has adopted a conformation allowing the presence of two cation-pi interaction between Arg 415 and the aromatic rings in addition to a hydrogen bond with the methoxy group (Fig. 7), three hydrogen bonds made by ser 508 and Arg 483 towards the methoxy groups, and another hydrogen bond between Leu 557 and NH_2_ group of the sulfonamide. Superimposition between compound **15** and the co-crystallized ligand showed that they adopt the same orientation inside the binding site (Fig. 8). Finally, compound **15** possessing the highest NQO1 inducer activity (CD = 20 μM) in this series showed the same interactions and the same orientation of the native ligand inside the receptor, indicating a possible correlation between those multiple interactions and the noted higher potency. Based on the above-mentioned results, compound **15** could possibly bind to Keap1 and disrupt its interaction with Nrf2.

The results from this study complement previous reports showing that the classical electrophilic Nrf2 activator sulforaphane protects cells, including human retinal pigment epithelial cells, keratinocytes, and mouse leukemia cells against oxidative damage caused by oxidative stressors of four different types, namely menadione, *tert*-butyl hydroperoxide, 4-hydroxynonenal, and peroxynitrite, as well as by exposure to ultraviolet radiation [65,66]. Furthermore, unlike the effects of most direct antioxidants, the indirect antioxidant effect of sulforaphane, which results from Nrf2 activation, persists for several days after sulforaphane is no longer present in the cell culture medium. This is because direct antioxidants, such as ascorbic acid, tocopherols, carotenoids, and polyphenols, which neutralize ROS and other chemical oxidants are consumed in these reactions, whereas Nrf2 activation results in transcriptional upregulation of antioxidant defences, which are mediated by proteins with long half-lives, often several days. The new compounds generated in the current study have an additional advantage in that they are non-electrophilic and are therefore expected to have a broader therapeutic window compared to electrophilic Nrf2 activators. This is supported by the very low toxicity of compound **15** in mice. Taken together, these results demonstrate the powerful effect of Nrf2 activation and induction of NQO1 in protecting cells and animals against high levels of ROS and preventing ROS-mediated damage. This is of particular relevance to protecting the hematopoietic system, which is highly sensitive to ROS.

## 3 Conclusion

In summary, a hybridization strategy was adopted using the iodinated quinazolinone scaffold and sulfonamide moiety to produce the 2-((6,8-diiodo-4-oxo-3-(4-sulfamoylphenyl)-3, 4-dihydroquinazolin-2-yl)thio)-*N*-(substituted) acetamide derivatives **5–18**. Different substitutions were introduced to the acetamide group to study the structure-activity relationship. All the compounds were screened for their antioxidant potential using the NQO1 inducer activity assay. The 3,4,5-trimethoxyphenyl derivative **15** showed the highest inducer activity in this series (CD = 20 μM) and had low toxicity (LD_50_ = 500 mg/kg). Treatment of gamma-irradiated mice with compound **15** lowered oxidative stress as evidenced by the lower levels of MDA, ROS and NQO1 in liver. Furthermore, compound **15** ameliorated the complete blood picture of irradiated mice, as well as enhanced the survival of mice over a period of 30 days post-irradiation. Molecular docking of **15** inside the active site of Keap1 confirmed that it binds in the same manner as that of the co-crystallized ligands. The inducer activity of compound **15** in upregulating NQO1 strongly suggests that it could be used as a lead antioxidant and radiomodulatory agent for further optimization of the quinazolinone scaffold.

## 4 Materials and methods

### 4.1 Chemistry

All chemicals were purchased from Sigma-Aldrich and are of AR grade. Melting points were determined in open capillary on a Gallen Kamp melting point apparatus (Sanyo Gallen Kamp, UK). Thin layer chromatography using precoated silica gel plates *(Kieselgel* 0.25 mm, 60 F254, Merck, Germany) was performed with a solvent system of chloroform/methanol (8:2) to detect the spots by UV light. IR spectra (KBr disc) were recorded using an FT-IR spectrophotometer (Perkin Elmer, USA). NMR spectra were scanned on NMR spectrophotometer (Bruker AXS Inc., Switzerland), operating at 500 MHz for ^1^H and 125.76 MHz for ^13^C. Mass spectra were recorded on the ISQ LT Thermo Scientific GCMS model (Massachusetts, USA). Chemical shifts are expressed in δ-values (ppm) relative to TMS as an internal standard, using DMSO‑*d*_6_ as a solvent. Elemental analyses were done on a model 2400 CHNSO analyser (Perkin Elmer, USA). All the values were within ±0.4% of the theoretical values.

#### 4.1.1 *4-(6, 8-diiodo-2-mercapto-4- oxoquinazolin-3(4H)-yl) benzenesulfonamide***(4)**

A mixture of 2-amino-3,5-diiodobenzoic acid **3** (3.88 g, 0.01 mol) and 4- isothiocyanatobenzenesulfonamide **2** (2.14 g, 0.01 mol) in absolute ethanol (30 mL) containing 3 drops of triethylamine, was refluxed for 3 h. The solid product formed was collected by filtration and crystallized from ethanol to give **4**.

**4:** Yield, 88%; m.p. > 300 °C. IR (KBr, ʋ cm^−1^): 3311, 3210 (NH_2_), 3098 (arom.), 1701 (CO), 1618 (CN), 1379, 1160 (SO_2_). ^1^H NMR (DMSO‑*d*_6_, *δ*, ppm): 1.97 (s, 1H), 7.86 (d, 2H, *J =* 7 Hz, AB), 8.06 (d, 2H, *J =* 7 Hz, AB), 8.23 (d, 1H, *J =* 2 Hz), 8.58 (d, 1H, *J =* 2 Hz), 10.05 (s, 2H). ^13^C NMR (DMSO‑*d*_6_, *δ*, ppm): 86.84, 89.74, 118.49, 118.84, 122.99, 127.24 (2), 133.61, 134.21, 136.20, 144.48, 158.31, 165.76, 176.14. Anal. Calcd. for C_14_H_9_I_2_N_3_O_3_S_2_ (584.82): C, 28.73; H, 1.55; N, 7.18. Found: C, 29.02; H, 1.82; N, 7.41.

#### 4.1.2 *3,4-Dihydroquinazolin-sulfonamide derivatives***(5-18)**

##### 4.1.2.1 General procedure

A mixture of **4** (5.85 g, 0.01 mol) and 2-chloro-*N*-substituted acetamide derivatives (0.01 mol) in dry acetone (30 mL) and anhydrous K_2_CO_3_ (1.38 g, 0.01 mol) was stirred at room temperature for 18 h, filtered and the solid product formed was crystallized from dioxane to give **5–18.**

##### 4.1.2.2 *2-((6,8-Diiodo-4-oxo-3-(4-sulfamoylphenyl)-3,4-dihydroquinazolin-*2-yl*)thio)-N-phenylacetamide***(5)**

**5:** Yield, 79%; m.p. > 300 °C. IR (KBr, ʋ cm^−1^): 3408, 3310, 3231 (NH, NH_2_), 3079 (arom.), 2945, 2881 (aliph.), 1702, 1679 (2CO), 1620 (CN), 1349, 1170 (SO_2_). ^1^H NMR (DMSO‑*d*_6_, *δ*, ppm): 4.17 (s, 2H), 7.03–7.30 (m, 3H), 7.60–7.83 (m, 4H), 8.02 (s, 2H), 8.09 (d, 2H, *J =* 10 Hz, AB), 8.35 (d, 1H, *J =* 2 Hz), 8.60 (d, 1H, *J =* 2 Hz), 10.12 (s, 1H). ^13^C NMR (DMSO‑*d*_6_, *δ*, ppm): 29.73, 81.27, 89.61, 118.79 (2), 123.62 (2), 124.33, 127.61, 128.05 (2), 128.80 (2), 133.12, 137.64, 137.90, 139.42, 145.13, 158.92, 163.31, 164.02, 169.81. MS *m/z* [%]: 718 [M^+^, 29.82], 719 [M+1, 19.85], 454 [100]. Anal. Calcd. for C_22_H_16_I_2_N_4_O_4_S_2_ (718.30): C, 36.78; H, 2.25; N, 7.80. Found: C, 37.01; H, 2.51; N, 8.12.

##### 4.1.2.3 *2-((6,8-Diiodo-4-oxo-3-(4-sulfamoylphenyl)-3,4-dihydroquinazolin-*2-yl*)thio)-N-o-tolylacetamide***(6)**

**6:** Yield, 81%; m.p. > 300 °C. IR (KBr, ʋ cm^−1^): 3403, 3321, 3216 (NH, NH_2_), 3098 (arom.), 2956, 2891 (aliph.), 1711, 1681 (2CO), 1631 (CN), 1355, 1188 (SO_2_). ^1^H NMR (DMSO‑*d*_6_, *δ*, ppm): 2.21 (s, 3H), 4.30 (s, 2H), 7.10 (ddd, 1H, *J =* 8 & 2.5 Hz), 7.30–7.55 (m, 3H), 7.87 (d, 2H, *J =* 8.5 Hz, AB), 8.01 (s, 2H), 8.05 (d, 2H, *J =* 8.5 Hz, AB), 8.28 (d, 1H, *J =* 1.5 Hz), 8.55 (d, 1H, *J =* 1.5 Hz), 9.66 (s, 1H). ^13^C NMR (DMSO‑*d*_6_, *δ*, ppm): 16.32, 26.61, 82.13, 91.41, 120.80 (2), 122.81, 123.90, 124.63, 128.72, 128.91 (2), 129.66, 130.81, 133.54, 134.02, 135.25, 135.87, 147.31, 153.82, 158.10, 159.62, 167.53. Anal. Calcd. for C_23_H_18_I_2_N_4_O_4_S_2_ (732.35): C, 37.72; H, 2.48; N, 7.65. Found: C, 38.04; H, 2.68; N, 7.96.

##### 4.1.2.4 *2-((6,8-Diiodo- 4-oxo-3-(4-sulfamoylphenyl)-3,4-dihydroquinazolin-*2-yl*)thio)-N-(m-tolyl)acetamide***(7)**

**7:** Yield, 86%; m.p. > 300 °C. IR (KBr, ʋ cm^−1^): 3421, 3318, 3207 (NH, NH_2_), 3095 (arom.), 2978, 2842 (aliph.), 1707, 1675 (2CO), 1625 (CN), 1378, 1145 (SO_2_). ^1^H NMR (DMSO‑*d*_6_, *δ*, ppm): 2.28 (s, 3H), 4.25 (s, 2H), 6.87 (m, 1H), 7.31–7.56 (m, 3H), 7.82 (d, 2H, *J* = 6.5 Hz, AB), 8.03 (d, 2H, *J* = 6.5 Hz, AB), 8.05 (s, 2H), 8.30 (d, 1H, *J* = 2 Hz), 8.50 (d, 1H, *J* = 2 Hz), 10.28 (s, 1H). ^13^C NMR (DMSO‑*d*_6_, *δ*, ppm): 24.13, 27.62, 84.64, 93.71, 117.10, 119.21, 120.63 (2), 122.81, 123.74, 127.62, 128.31 (2), 133.04, 134.22, 135.41, 137.15, 137.90, 146.33, 153.91, 158.03, 161.85, 169.63. Anal. Calcd. for C_23_H_18_I_2_N_4_O_4_S_2_ (732.35): C, 37.72; H, 2.48; N, 7.65. Found: C, 38.07; H, 2.72; N, 7.98.

##### 4.1.2.5 *2-((6,8-Diiodo-4-oxo-3-(4-sulfamoylphenyl)-3,4-dihydroquinazolin-*2-yl*)thio)-N-(p-tolyl)acetamide***(8)**

**8:** Yield, 85%; m.p. > 300 °C. IR (KBr, ʋ cm^−1^): 3419, 3331, 3197 (NH, NH_2_), 3089 (arom.), 2995, 2852 (aliph.), 1710, 1682 (2CO), 1623 (CN), 1391, 1161 (SO_2_). ^1^H NMR (DMSO‑*d*_6_, *δ*, ppm): 2.30 (s, 3H), 4.24 (s, 2H), 7.11 (d, 2H, *J* = 6 Hz, AB), 7.61 (m, 2H), 7.90 (d, 2H, *J* = 8 Hz, AB), 8.04 (d, 2H, *J* = 7 Hz, AB), 8.05 (s, 2H), 8.37 (d, 1H, *J* = 2 Hz), 8.58 (d, 1H, *J* = 2 Hz), 10.43 (s, 1H). ^13^C NMR (DMSO‑*d*_6_, *δ*, ppm): 19.21, 25.64, 80.92, 94.07, 119.21 (2), 120.16 (2), 123.72, 128.35 (2), 129.12 (2), 132.73, 134.61, 134.90, 135.11, 135.85, 145.62, 154.57, 160.61, 161.43, 165.90. MS *m/z* [%]: 733 [M^+^, 68.30], 681 [100]. Anal. Calcd. for C_23_H_18_I_2_N_4_O_4_S_2_ (732.35): C, 37.72; H, 2.48; N, 7.65. Found: C, 38.11; H, 2.80; N, 8.02.

##### 4.1.2.6 *2-((6,8-Diiodo-4-oxo-3-(4-sulfamoylphenyl)-3,4-dihydroquinazolin-*2-yl*)thio)-N-(2-ethylphenyl) acetamide***(9)**

**9:** Yield, 79%; m.p. > 300 °C. IR (KBr, ʋ cm^−1^): 3402, 3321, 3181 (NH, NH_2_), 3092 (arom.), 2982, 2860 (aliph.), 1720, 1685 (2CO), 1619 (CN), 1387, 1142 (SO_2_). ^1^H NMR (DMSO‑*d*_6_, *δ*, ppm): 1.31 (t, 3H, *J =* 15 Hz), 2.54 (q, 2H, *J =* 12 Hz), 4.25 (s, 2H), 7.03 (dd, 1H, *J* = 8 & 2 Hz), 7.15 (m, 1H), 7.25 (ddd, 1H, *J* = 7 & 6 Hz), 7.30 (dd, 1H, *J* = 7 & 2 Hz), 7.76 (d, 2H, *J* = 9 Hz, AB), 8.03 (d, 2H, *J* = 7.5 Hz, AB), 8.04 (m, 2H), 8.32 (d, 1H, *J* = 2.5 Hz), 8.61 (d, 1H, *J* = 2.5 Hz), 9.79 (s, 1H). ^13^C NMR (DMSO‑*d*_6_, *δ*, ppm): 14.67, 24.23, 37.14, 91.81, 101.47, 121.87 (2), 126.36, 126.47, 126.67, 127.59, 128.88, 130.59 (2), 135.59, 135.74, 138.65, 139.69 (2), 146.65, 151.48, 158.80, 159.67, 165.71. MS *m/z* [%]: 746 [M^+^, 26.98], 747 [M+1, 5.94], 368 [100]. Anal. Calcd. for C_24_H_20_I_2_N_4_O_4_S_2_ (746.38): C, 38.62; H, 2.70; N, 7.51. Found: C, 39.01; H, 3.01; N, 7.78.

##### 4.1.2.7 *2-((6,8-Diiodo-4-oxo-3-(4-sulfamoylphenyl)-3,4-dihydroquinazolin-2-yl)thio)-N-(3-ethylphenyl) acetamide***(10)**

**10:** Yield, 75%; m.p. > 300 °C. IR (KBr, ʋ cm^−1^): 3412, 3313, 3192 (NH, NH_2_), 3085 (arom.), 2994, 2853 (aliph.), 1714, 1679 (2CO), 1622 (CN), 1393, 1157 (SO_2_). ^1^H NMR (DMSO‑*d*_6_, *δ*, ppm): 1.20 (t, 3H, *J =* 15 Hz), 2.58 (q, 2H, *J =* 12 Hz), 4.21 (s, 2H), 6.98 (ddd, 1H, *J* = 8 & 2 Hz), 7.21–7.48 (m, 2H), 7.70–8.04 (m, 5H), 8.05 (s, 2H), 8.23 (d, 1H, *J* = 3 Hz), 8.54 (d, 1H, *J* = 3 Hz), 10.32 (s, 1H). ^13^C NMR (DMSO‑*d*_6_, *δ*, ppm): 15.21, 24.10, 29.82, 82.60, 92.01, 120.43 (2), 122.71, 128.58, 125.73, 126.02, 126.68, 128.81, 129.12 (2), 133.65, 134.01, 135.28, 135.91, 144.80, 156.02, 160.51, 160.95, 168.12. Anal. Calcd. for C_24_H_20_I_2_N_4_O_4_S_2_ (746.38): C, 38.62; H, 2.70; N, 7.51. Found: C, 38.31; H, 2.44; N, 7.20.

##### 4.1.2.8 *2-((6,8-Diiodo-4-oxo-3-(4-sulfamoylphenyl)-3,4-dihydroquinazolin-2-yl)thio)-N-(4-ethylphenyl) acetamide***(11)**

**11:** Yield, 73%; m.p. > 300 °C. IR (KBr, ʋ cm^−1^): 3422, 3330, 3201 (NH, NH_2_), 3094 (arom.), 2986, 2847 (aliph.), 1710, 1678 (2CO), 1631 (CN), 1384, 1156 (SO_2_). ^1^H NMR (DMSO‑*d*_6_, *δ*, ppm): 1.15 (t, 3H, *J =* 10 Hz), 2.55 (q, 2H, *J =* 8 Hz), 4.23 (s, 2H), 7.12 (d, 2H, *J* = 9 Hz, AB), 7.61 (d, 2H, *J* = 9 Hz, AB), 7.79 (d, 2H, *J* = 8.5 Hz), 7.80–8.05 (m, 4H), 8.39 (d, 1H, *J* = 2 Hz), 8.58 (d, 1H, *J* = 2 Hz), 10.31 (s, 1H). ^13^C NMR (DMSO‑*d*_6_, *δ*, ppm): 17.12, 29.40, 29.62, 83.25, 93.04, 120.61 (2), 120.83 (2), 123.10, 127.65 (2), 128.02 (2), 133.71, 135.07, 135.63, 135.80, 140.92, 150.13, 153.81, 160.72, 160.97, 166.32. Anal. Calcd. for C_24_H_20_I_2_N_4_O_4_S_2_ (746.38): C, 38.62; H, 2.70; N, 7.51. Found: C, 38.97; H, 2.96; N, 7.78.

##### 4.1.2.9 *2-((6,8-Diiodo- 4-oxo-3-(4-sulfamoylphenyl)-3, 4-dihydroquinazolin-2-yl)thio)-N -(4-methoxyphenyl) acetamide***(12)**

**12:** Yield, 79%; m.p. > 300 °C. IR (KBr, ʋ cm^−1^): 3397, 3325, 3194 (NH, NH_2_), 3087 (arom.), 2990, 2852 (aliph.), 1716, 1684 (2CO), 1627 (CN), 1396, 1140 (SO_2_). ^1^H NMR (DMSO‑*d*_6_, *δ*, ppm): 3.75 (s, 3H), 4.29 (s, 2H), 6.87 (d, 2H, *J* = 9 Hz, AB), 7.56 (d, 2H, *J* = 9 Hz, AB), 7.78 (d, 2H, *J* = 7 Hz, AB), 7.95 (d, 2H, *J* = 7 Hz), 8.04 (s, 2H), 8.27 (d, 1H, *J* = 2.5 Hz), 8.61 (d, 1H, *J* = 2.5 Hz), 10.20 (s, 1H). ^13^C NMR (DMSO‑*d*_6_, *δ*, ppm): 29.43, 54.26, 83.03, 91.67, 112.01 (2), 118.33 (2), 123.14 (2), 123.85, 127.82 (2), 129.64, 134.12, 134.83, 135.90, 150.23, 156.71, 157.42, 160.04, 161.62, 169.31. Anal. Calcd. for C_23_H_18_I_2_N_4_O_5_S_2_ (748.35): C, 36.91; H, 2.42; N, 7.49. Found: C, 37.27; H, 2.70; N, 7.82.

##### 4.1.2.10 *2-((6,8-Diiodo-4-oxo-3-(4-sulfamoylphenyl)-3,4-dihydroquinazolin-2-yl)thio)-N-(4-ethoxyphenyl) acetamide***(13)**

**13:** Yield, 78%; m.p. > 300 °C. IR (KBr, ʋ cm^−1^): 3385, 3295, 3188 (NH, NH_2_), 3096 (arom.), 2979, 2844 (aliph.), 1705, 1680 (2CO), 1608 (CN), 1387, 1148 (SO_2_). ^1^H NMR (DMSO‑*d*_6_, *δ*, ppm): 1.27 (t, 3H, *J =* 12 Hz), 3.97 (q, 2H, *J =* 10 Hz), 4.21 (s, 2H), 6.89 (d, 2H, *J* = 10 Hz, AB), 7.39 (d, 2H, *J* = 10 Hz, AB), 7.76 (d, 2H, *J* = 8 Hz, AB), 8.03–8.10 (m, 4H), 8.29 (d, 1H, *J* = 2 Hz), 8.62 (d, 1H, *J =* 2 Hz), 10.29 (s, 1H). ^13^C NMR (DMSO‑*d*_6_, *δ*, ppm): 15.21, 29.63, 62.04, 83.72, 92.57, 115.03 (2), 120.61 (2), 121.40 (2), 122.74, 127.66 (2), 129.52, 134.67, 134.91, 135.78, 146.83, 154.02, 155.25, 160.12, 162.46, 169.60. Anal. Calcd. for C_24_H_20_I_2_N_4_O_5_S_2_ (762.38): C, 37.81; H, 2.64; N, 7.35. Found: C, 38.20; H, 3.00; N, 7.65.

##### 4.1.2.11 *2-((6,8-Diiodo-4-oxo-3-(4-sulfamoylphenyl)-3,4-dihydroquinazolin-2-yl)thio)-N-(3,5-dimethoxyphenyl) acetamide***(14)**

**14:** Yield, 67%; m.p. > 300 °C. IR (KBr, ʋ cm^−1^): 3256, 3209, 3142 (NH, NH_2_), 3065 (arom.), 2912, 2844 (aliph.), 1676, 1663 (2CO), 1608 (CN), 1385, 1161 (SO_2_). ^1^H NMR (DMSO‑*d*_6_, *δ*, ppm): 3.74 (s, 6H), 4.21 (s, 2H), 6.25 (dd, 1H, *J* = 3 & 1.5 Hz), 6.87 (dd, 2H, *J* = 3 & 1.5 Hz), 7.77 (d, 2H, *J* = 9 Hz, AB), 8.04 (d, 2H, *J* = 9 Hz, AB), 8.05 (s, 2H), 8.34 (d, 1H, *J* = 2.5 Hz), 8.63 (d, 1H, *J* = 2.5 Hz), 10.38 (s, 1H). ^13^C NMR (DMSO‑*d*_6_, *δ*, ppm): 31.32, 55.56 (2), 91.66, 95.96, 98.12, 101.11 (2), 121.92 (2), 127.61, 130.64 (2), 135.64, 138.77 (2), 140.93, 146.14, 151.43, 158.86, 159.59 (2), 160.90, 165.20. Anal. Calcd. for C_24_H_20_I_2_N_4_O_6_S_2_ (778.38): C, 37.03; H, 2.59; N, 7.20. Found: C, 37.34; H, 2.83; N, 7.43.

##### 4.1.2.12 *2-((6,8-Diiodo-4-oxo-3-(4-sulfamoylphenyl)-3,4-dihydroquinazolin-2-yl)thio)-N-(3,4,5-trimethoxyphenyl) acetamide***(15)**

**15:** Yield, 80%; m.p. > 300 °C. IR (KBr, ʋ cm^−1^): 3416, 3319, 3200 (NH, NH_2_), 3093 (arom.), 2982, 2822 (aliph.), 1689, 1666 (2CO), 1616 (CN), 1370, 1151 (SO_2_). ^1^H NMR (DMSO‑*d*_6_, *δ*, ppm): 3.70 (s, 6H), 3.81 (s, 3H), 4.31 (s, 2H), 6.90 (d, 2H*, J* = 3 Hz), 7.78 (d, 2H, *J* = 9 Hz, AB), 8.04 (s, 2H), 8.05 (d, 2H, *J* = 9 Hz), 8.28 (d, 1H, *J* = 2.5 Hz, AB), 8.54 (d, 1H, *J* = 2.5 Hz), 10.36 (s, 1H). ^13^C NMR (DMSO‑*d*_6_, *δ*, ppm): 27.23, 55.60 (2), 61.32, 82.74, 95.07, 101.41 (2), 119.60 (2), 121.82, 130.74 (2), 131.90, 133.81, 134.15, 134.67, 134.92, 146.04, 151.32 (2), 153.50, 160.01, 160.73, 166.37. MS *m/z* [%]: 809 [M^+^, 15.75], 456 [100]. Anal. Calcd. for C_25_H_22_I_2_N_4_O_7_S_2_ (808.40): C, 37.14; H, 2.74; N, 6.93. Found: C, 37.44; H, 3.03; N, 7.29.

##### 4.1.2.13 *2-((6,8-Diiodo-4-oxo-3-(4-sulfamoylphenyl)-3,4-dihydroquinazolin-2-yl)thio)-N-(2-methyl-4-nitrophenyl) acetamide***(16)**

**16:** Yield, 74%; m.p. > 300 °C. IR (KBr, ʋ cm^−1^): 3313, 3265, 3203 (NH, NH_2_), 3055 (arom.), 2943, 2811 (aliph.), 1683, 1660 (2CO), 1621 (CN), 1530, 1370 (NO_2_), 1397, 1155 (SO_2_). ^1^H NMR (DMSO‑*d*_6_, *δ*, ppm): 2.39 (s, 3H), 4.30 (s, 2H), 7.72 (d, 1H, *J* = 9.5 Hz), 7.88 (d, 2H, *J* = 8 Hz, AB), 7.90–8.05 (m, 6H), 8.26 (d, 1H, *J* = 3 Hz), 8.59 (d, 1H, *J* = 3 Hz), 10.27 (s, 1H). ^13^C NMR (DMSO‑*d*_6_, *δ*, ppm): 18.05, 27.34, 83.69, 92.03, 106.32, 120.85, 121.71 (2), 125.83, 127.61, 130.64 (2), 133.72, 134.80, 134.92, 135.01, 141.42, 141.94, 147.56, 154.02, 160.82, 161.04, 167.42. Anal. Calcd. for C_23_H_17_I_2_N_5_O_6_S_2_ (777.35): C, 35.54; H, 2.20; N, 9.01. Found: C, 35.78; H, 2.57; N, 9.32.

##### 4.1.2.14 *2-((6, 8-Diiodo-4-oxo-3-(4-sulfamoylphenyl)-3, 4-dihydroquinazolin-2-yl)thio)- N-(2-methyl-6-nitrophenyl) acetamide***(17)**

**17:** Yield, 70%; m.p. > 300 °C. IR (KBr, ʋ cm^−1^): 3329, 3283, 3174 (NH, NH_2_), 3087 (arom.), 2952, 2828 (aliph.), 1680, 1662 (2CO), 1615 (CN), 1545, 1340 (NO_2_), 1376, 1155 (SO_2_). ^1^H NMR (DMSO‑*d*_6_, *δ*, ppm): 2.21 (s, 3H), 4.27 (s, 2H), 7.34 (dd, 1H, *J* = 9 & 6 Hz), 7.76 (dd, 1H, *J* = 9 & 2.5 Hz), 7.82 (d, 2H, *J* = 10 Hz), 8.01–8.10 (m, 5H), 8.27 (d, 1H, *J* = 2.5 Hz), 8.67 (d, 1H, *J* = 2.5 Hz), 10.02 (s, 1H). ^13^C NMR (DMSO‑*d*_6_, *δ*, ppm): 18.17, 36.90, 91.83, 101.40, 121.87 (2), 122.70, 127.43, 127.63, 128.79, 130.67 (2), 135.49, 135.62 (2), 137.85 (2), 138.68, 146.93, 151.50, 158.34, 159.67, 165.87. MS *m/z* [%]: 777 [M^+^, 4.30], 64 [100]. Anal. Calcd. for C_23_H_17_I_2_N_5_O_6_S_2_ (777.35): C, 35.54; H, 2.20; N, 9.01. Found: C, 35.80; H, 2.47; N, 9.33.

##### 4.1.2.15 *2-((6, 8-Diiodo-4-oxo-3-(4-sulfamoylphenyl)-3, 4-dihydroquinazolin-2-yl)thio)- N-(2,4-dinitrophenyl) acetamide***(18)**

**18:** Yield, 66%; m.p. > 300 °C. IR (KBr, ʋ cm^−1^): 3432, 3303, 3165 (NH, NH_2_), 3058 (arom.), 2961, 2850 (aliph.), 1690, 1683 (2CO), 1608 (CN), 1537, 1361 (NO_2_), 1388, 1161 (SO_2_). ^1^H NMR (DMSO‑*d*_6_, *δ*, ppm): 4.31 (s, 2H), 7.75 (d, 2H, *J* = 8 Hz, AB), 7.90 (d, 1H, *J* = 10 Hz), 8.00–8.04 (m, 2H), 8.04 (d, 2H, *J =* 8 Hz, AB), 8.29 (d, 1H, *J* = 2.5 Hz), 8.30–8.34 (m, 3H), 11.21 (s, 1H). ^13^C NMR (DMSO‑*d*_6_, *δ*, ppm): 29.21, 86.23, 102.13, 117.69, 121.03 (2), 122.12, 124.25, 127.43 (2), 131.16, 134.64 (2), 139.50 (2), 142.62, 144.12, 149.04, 154.91, 160.24, 165.65, 167.45. Anal. Calcd. for C_22_H_14_I_2_N_6_O_8_S_2_ (808.32): C, 32.69; H, 1.75; N, 10.40. Found: C, 32.36; H, 1.47; N, 10.18.

### 4.2 Biological evaluation

#### 4.2.1 NQO1 *in vitro* inducer activity

Hepa1c1c7 murine hepatoma cells were grown in a humidified atmosphere at 37 °C, 5% CO_2_. The cells were tested routinely to ensure that they were mycoplasma-free. The α-minimum essential medium (α-MEM) supplemented with 10% (v/v) heat- and charcoal-inactivated (1 g/100 mL, 90 min at 55 °C) fetal bovine serum was used. For evaluation of the potential NQO1 inducer activity, cells (10^4^/well) were grown in transparent flat-bottom plastic 96-well plates for 24 h, after which the cell culture medium was replaced with fresh medium containing each inducer (dissolved in DMSO and diluted in the medium 1:1000), and the cells were grown for further 48 h. Three replicates of each treatment of eight serial dilutions of inducers were used. The final DMSO concentration in the cell culture medium was maintained at 0.1% (v/v) in all wells. Cell lysates were prepared in digitonin and the specific activity of NQO1 was determined using menadione as a substrate as described [67,68]. Briefly, the cell culture medium was removed from each well, and the cells were washed three times with 200 μL of phosphate buffered saline (PBS), and subsequently lysed in 75 μL of digitonin suspension in the presence of EDTA for 20 min with shaking. Of the cell lysate, 20 μL was transferred to the well of a new plate and used to determine the protein concentration by adding 300 μL of bicinchoninic acid reagent and measuring the reaction product spectrophotometrically in a 96-well plate reader at 550 nm after 30 min incubation at room temperature. The remaining 55 μL of the cell lysate was used to measure the enzyme activity of NQO1. This was done by addition of 200 μL of enzyme assay buffer, containing NADPH-generating system (glucose-6-phosphate, glucose-6-phosphate dehydrogenase, NADP) that maintained a constant NADPH concentration, FAD, menadione (2-methyl-1,4-naphthoquinone, a quinone that is reduced to menadiol by NQO1 in the presence of NADPH), and MTT (3-[4,5-dimethylthiazo-2-yl]-2,5-diphenyltetrazolium bromide; a tetrazolium dye that is reduced non-enzymatically to a formazan dye by menadiol). The reaction was terminated after 5 min by the addition of dicumarol (a potent inhibitor of NQO1), and the reduced formazan dye was measured spectrophotometrically at 610 nm. The Concentration that Doubles the specific activity of NQO1 (CD value) was used as a measure of inducer potency. Mean values for the eight replicate wells are shown for each data point. The standard deviation for each data point was within 5% of the mean value. The classical NQO1 inducer sulforaphane was included as a positive control in each bioassay and was consistently giving a CD value of 0.2 μM.

#### 4.2.2 In vivo evaluation

##### 4.2.2.1 Animals

Eight-week old Swiss albino male mice (20–25 g) were supplied from the breeding unit of the National Center for Radiation Research and Technology (NCRRT), Cairo, Egypt. They were housed in the laboratory room for one week prior to the experiment for acclimatization to the lab environment. Water and food were allowed ad libitum. Mice were kept under controlled conditions: room temperature of 25 ± 5 °C, humidity (60 ± 5%), alternating 12 h dark and 12 h light cycle. Animals were treated gently; squeezing, pressure and tough handling were avoided. All animal procedures were performed in accordance with the Ethics Committee for Animal Experimentations, Faculty of Pharmacy, Cairo University, which complies with the Guide for the Care and Use of Laboratory Animals issued by the US National Institutes of Health (NIH Publication No. 85-23, revised 2011).

##### 4.2.2.2 Irradiation process

Mice were exposed to whole-body gamma radiation as a single dose of 7 Gy using Canadian Gamma Cell-40 biological irradiator (^137^Cs) located at the NCRRT, Cairo, Egypt and the dose rate was 0.655 rad/s.

##### 4.2.2.3 Acute toxicity study

The median lethal dose (LD_50_) of the most promising compound, **15** was determined according to Chinedu *et al.* [69].

##### 4.2.2.4 Experimental design

Eighty mice were randomly classified into four groups. First (control) group was injected i.p. with 10% DMSO, daily, for 5 days. Second (irradiated) group was treated as control, and after 1 h from last DMSO injection, the mice were irradiated at a dose of 7 Gy. Third (Compound **15**) group; received 50 mg/kg/day i.p. (1/10 LD_50_) of compound **15**, daily for 5 days. Fourth (Compound **15** + Irradiation) group was treated as third group then on the last day, after 1 h of injection, mice exposed to 7 Gy gamma radiation. On the third day, five mice from each group (n = 5) were weighed and anesthetized using urethane (1.2 mg/kg i.p) [70]. Then the blood samples were collected by cardiac puncture. At that time, they were euthanized by cervical dislocation. Each blood sample was collected into EDTA coated tubes for complete blood picture. Liver tissues were rinsed with ice-cold saline, dried on a filter paper and weighed to calculate the relative liver/body weight ratio. Then, it was homogenized in ice-cold 0.1 M phosphate buffer saline (pH 7.4) and stored at −80 °C till used for subsequent biochemical analysis. The residual of mice in all the groups was monitored on a daily basis for 30 days to check the survival rate, as well as their body weight, were recorded weekly to estimate the changes in body weight.

##### 4.2.2.5 Biochemical parameters investigated in liver homogenate

Liver homogenates were used for measuring the level of Nrf2 using colorimetric cell-based Elisa kit (OKAG00918) Aviva systems biology (San Diego, CA., USA), as well as the level of NAD(P)H: quinone oxidoreductase 1 (NQO1) was measured using an ELISA Kit (OKCD02727) Aviva Systems Biology (San Diego, CA., USA). Liver lipid peroxides were determined by measuring MDA as an indicator according to the method of Yoshioka *et al.* [71]. The generation of ROS in liver tissues was measured according to a modified technique of Vrablic *et al.* [72].

##### 4.2.2.6 Hematological analysis

Complete blood count with platelet count was determined using the automated micro-analyzer (BC-2800 Mindray, China).

##### 4.2.2.7 Statistical analysis

Data were analyzed using Prism 5.03 (GraphPad, San Diego, CA, USA) and expressed as means ± standard error. Comparisons between groups were analyzed by one-way analysis of variance (ANOVA) followed by Bonferroni’s multiple comparison test. Survival was analyzed by the Kaplan–Meier method followed by the Mantel–Cox (log rank) test. Body weight changes between groups through 30 days were analyzed by two-way ANOVA followed by Bonferroni’s post test. *P* < 0.05 was considered to represent statistically significant differences.

### 4.3 Molecular docking

The molecular modeling studies were fulfilled by the Molecular Operating Environment software (MOE, 10.2008). The receptor was chosen from the protein data bank; 4IQK that represents Keap1 co-crystallized with *N,N′*-naphthalene-1,4-diylbis(4-methoxybenzenesulfonamide. The protein was prepared for docking by ignoring water in the receptor. Hydrogen atoms were added to the structure with their standard geometry. The co-crystallized ligand was used to determine the binding site. Triangle Matcher placement method and dG scoring function were used for docking. Energy minimizations were performed with an RMSD gradient of 0.1 kcal mol^−1^Å^−1^with the MMFF94X force field and the partial charges were automatically calculated. Validation of the docking protocol was performed by re-docking of the co-crystallized ligands into the active site of Keap1 protein followed by docking of compound **15**. The obtained data were used to interpret the ligand-protein interactions at the Nrf2-binding site.

## Declaration of competing interest

The authors declare that they have no known competing financial interests or personal relationships that could have appeared to influence the work reported in this paper.
