# Supplementary figures and images for: Radiomodulatory effect of a non-electrophilic NQO1 inducer identified in a screen of new 6, 8-diiodoquinazolin-4(3H)-ones carrying a sulfonamide moiety
Source: Eur J Med Chem. 2020 Aug 15;200:112467. doi: 10.1016/j.ejmech.2020.112467 (PMC7355233; doi:10.1016/j.ejmech.2020.112467)

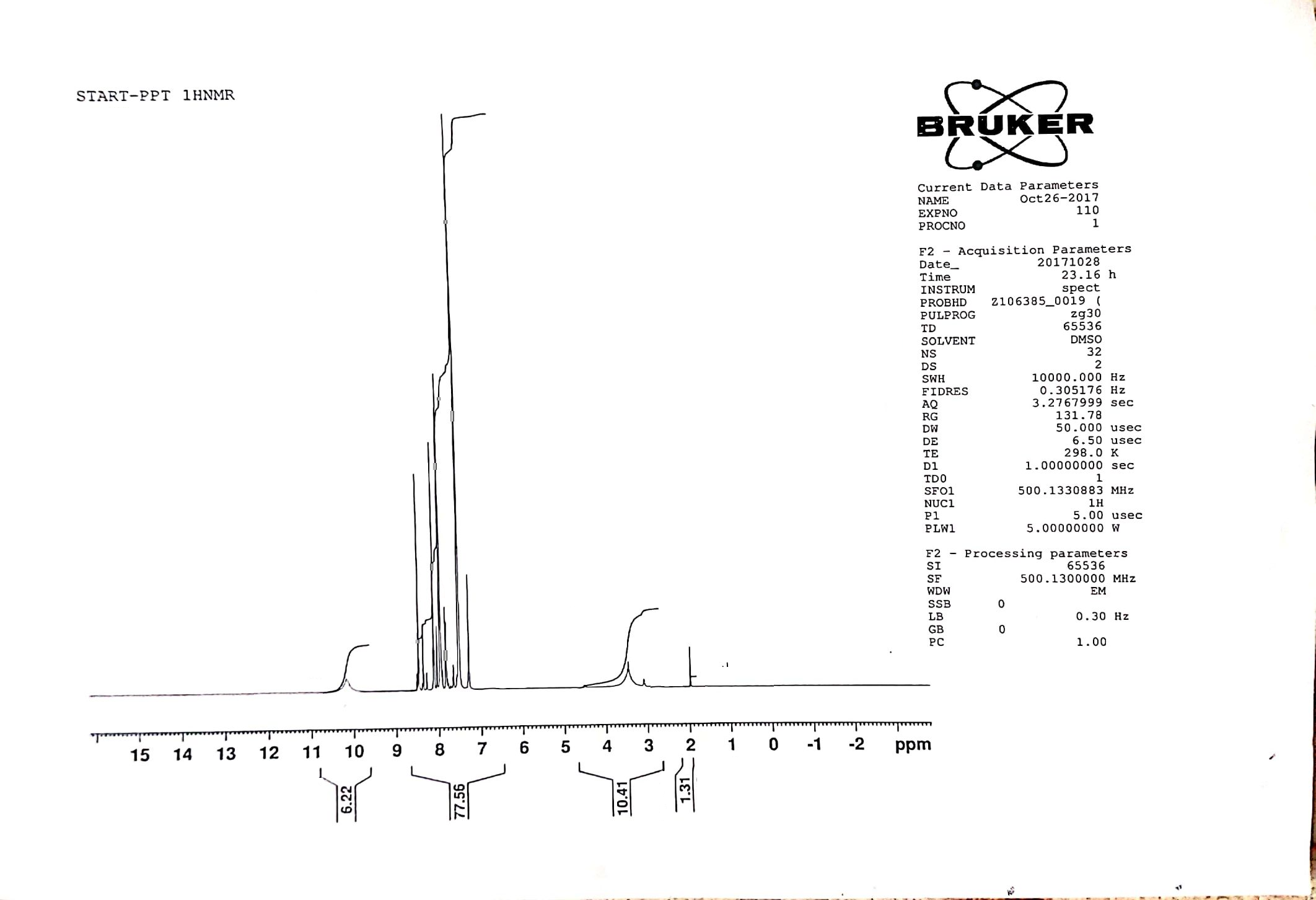


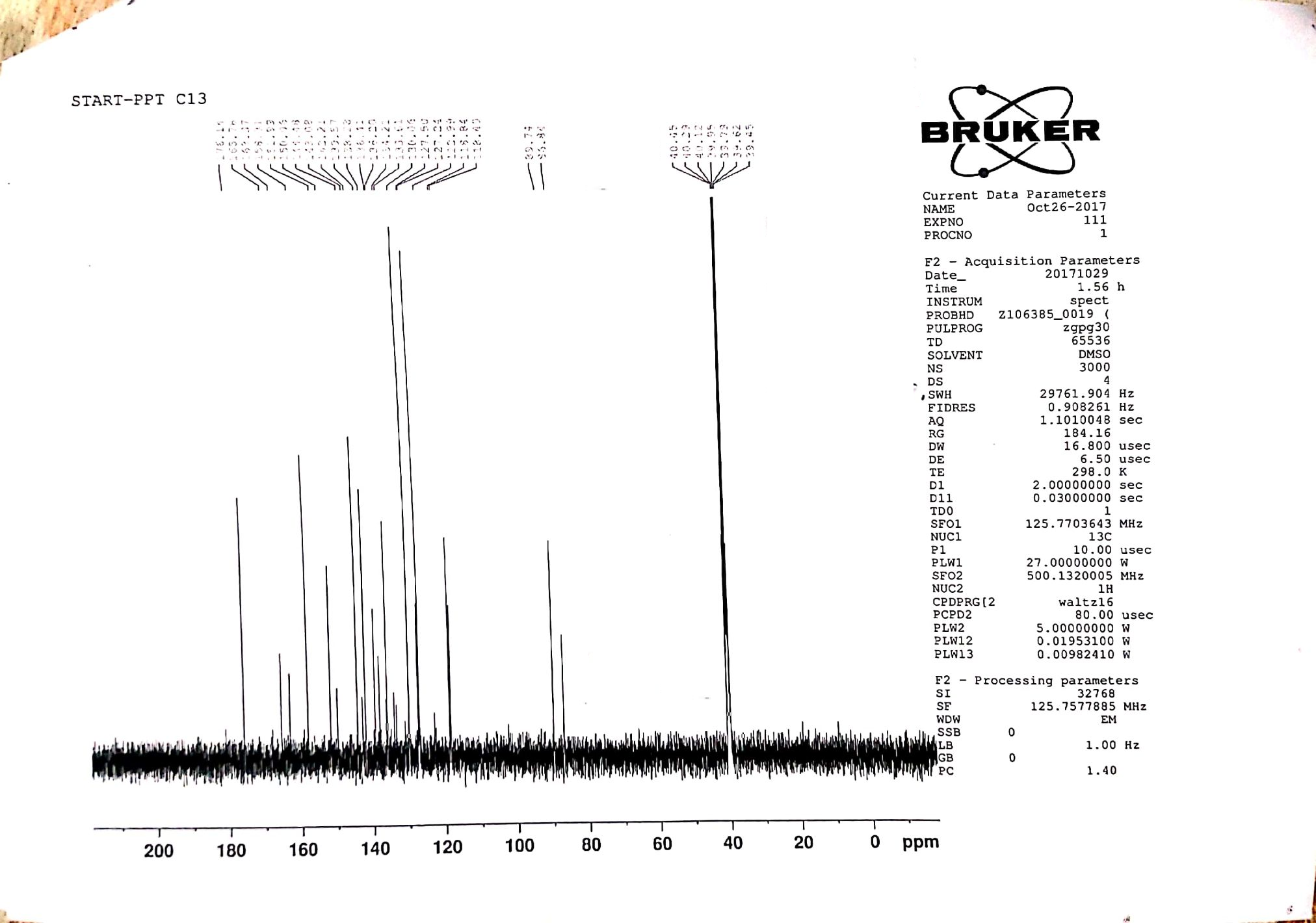


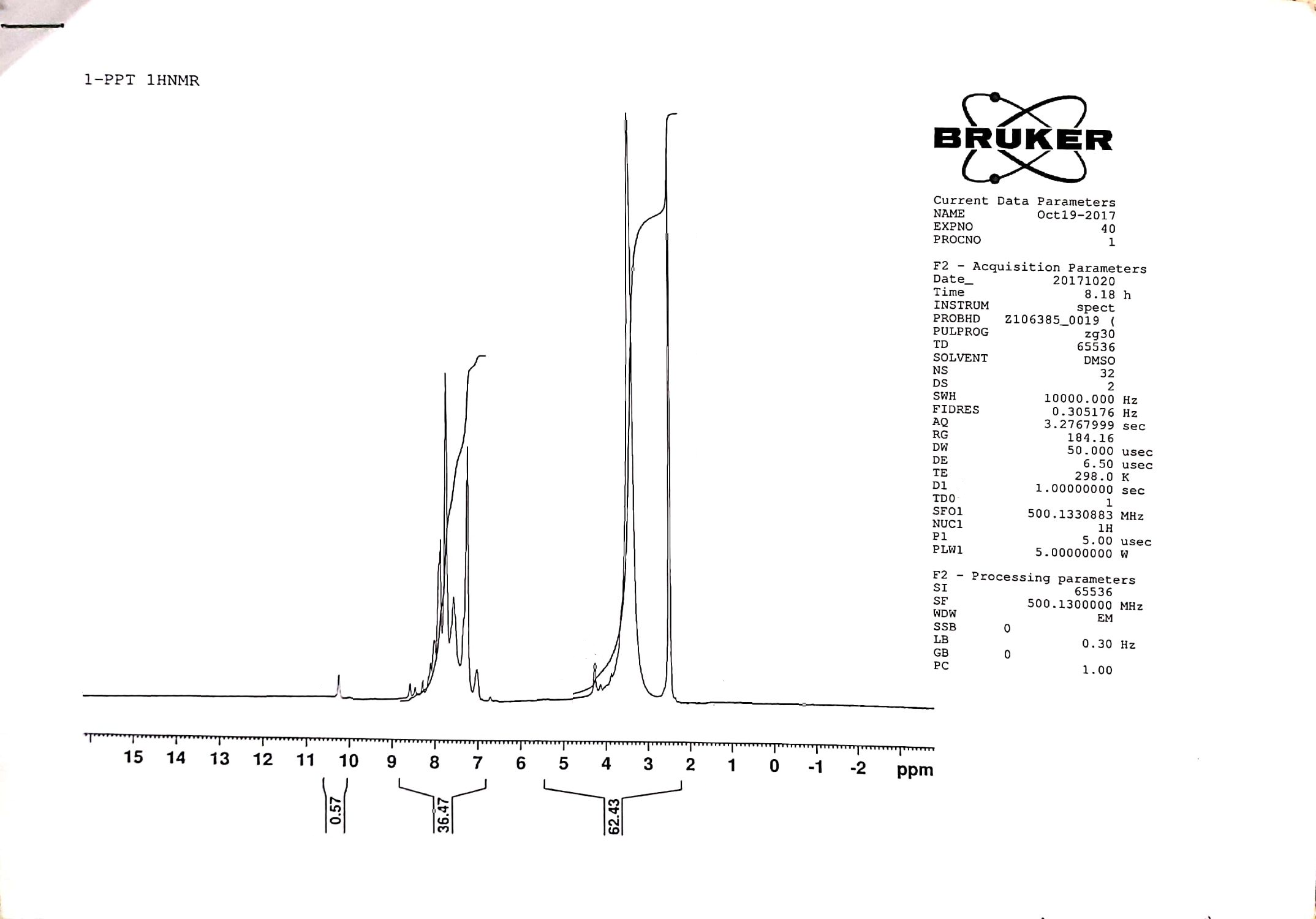


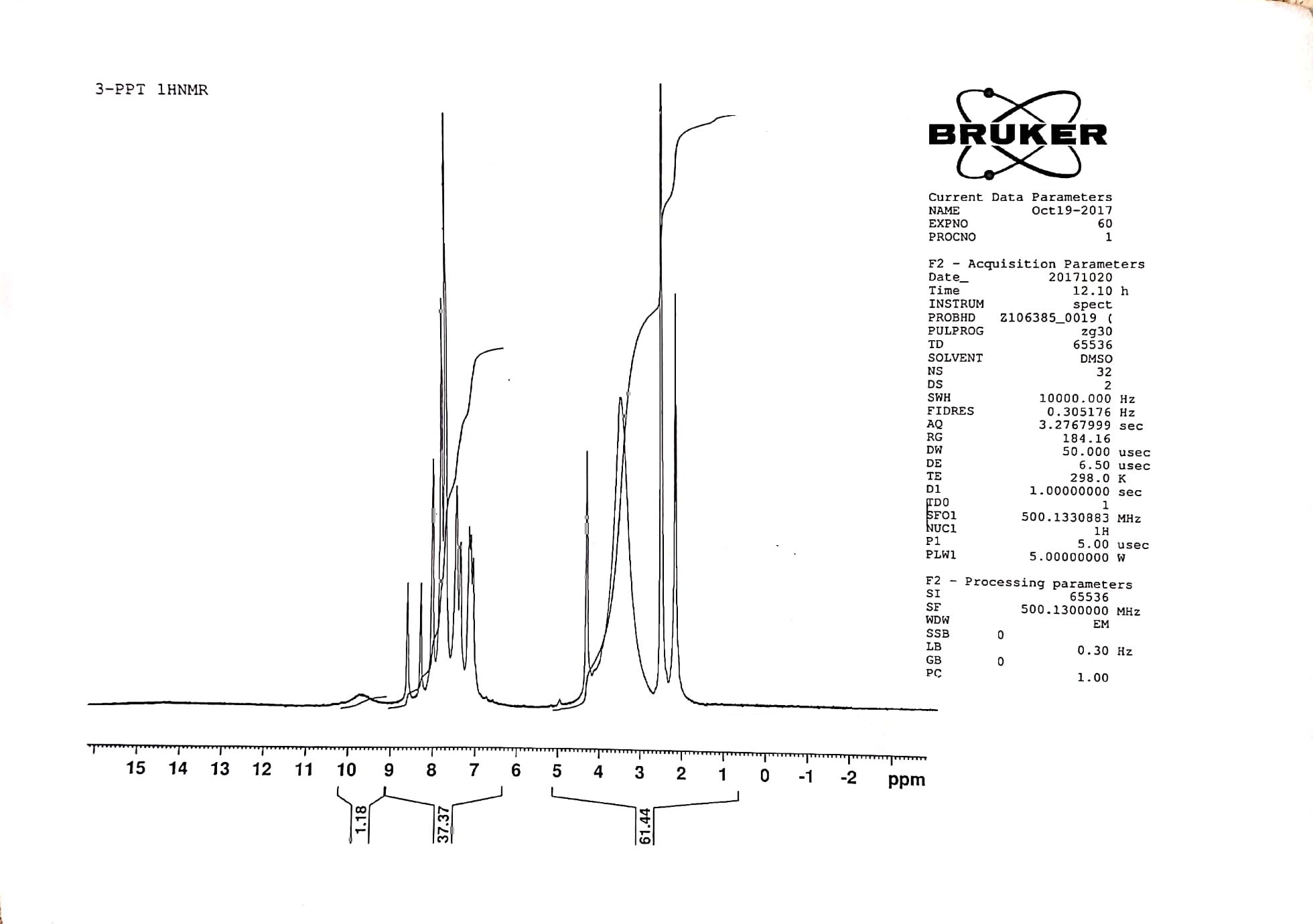


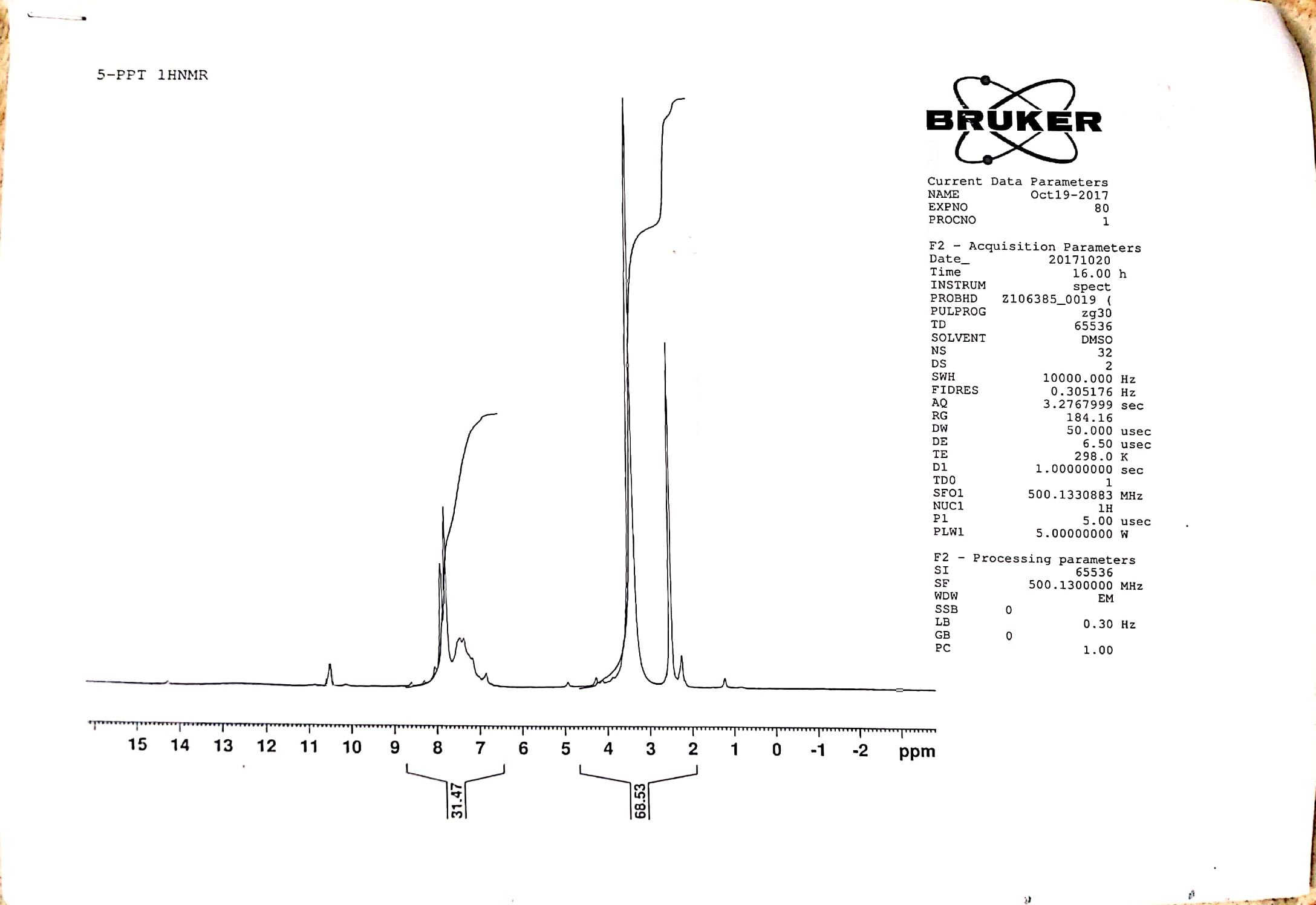


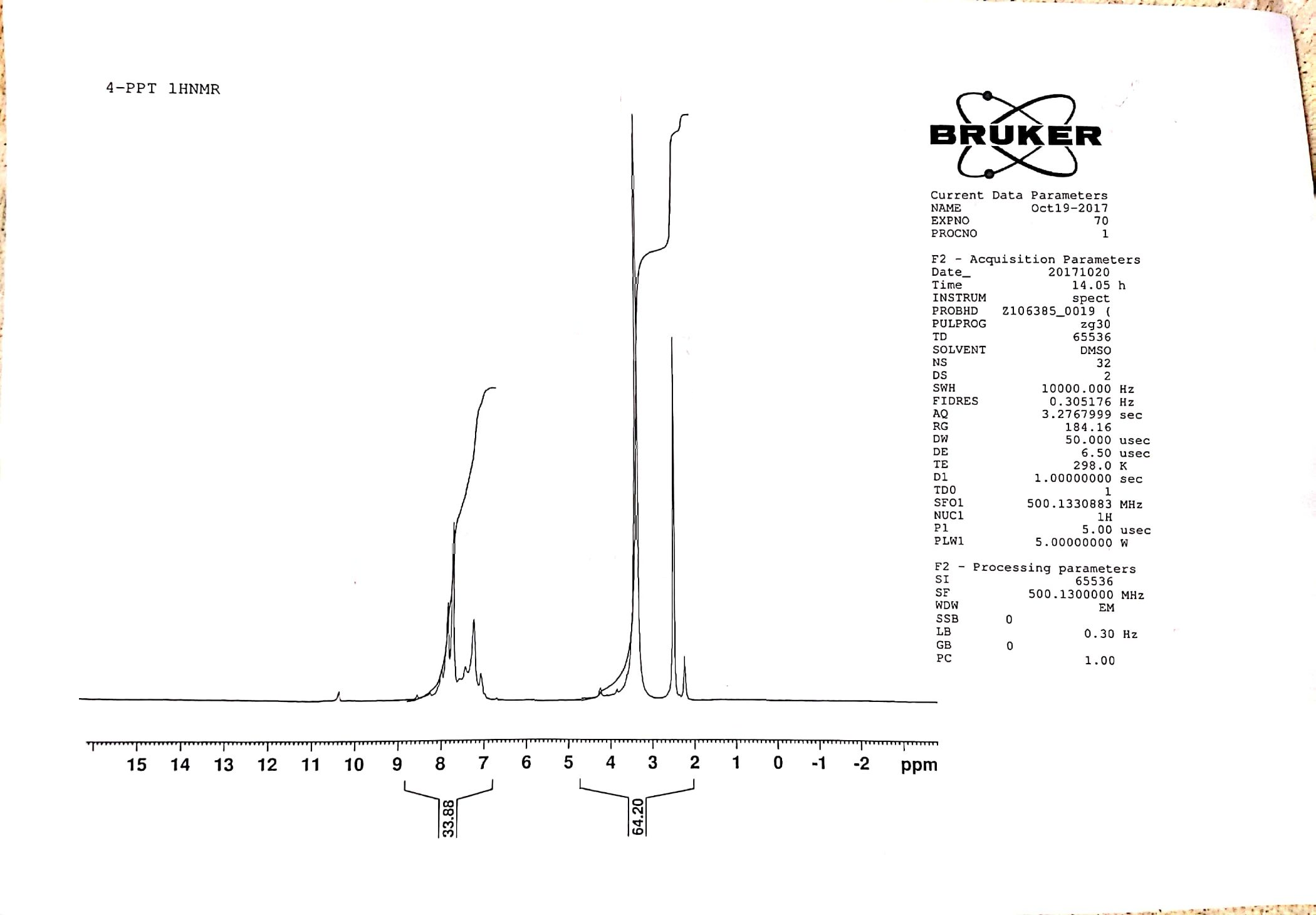


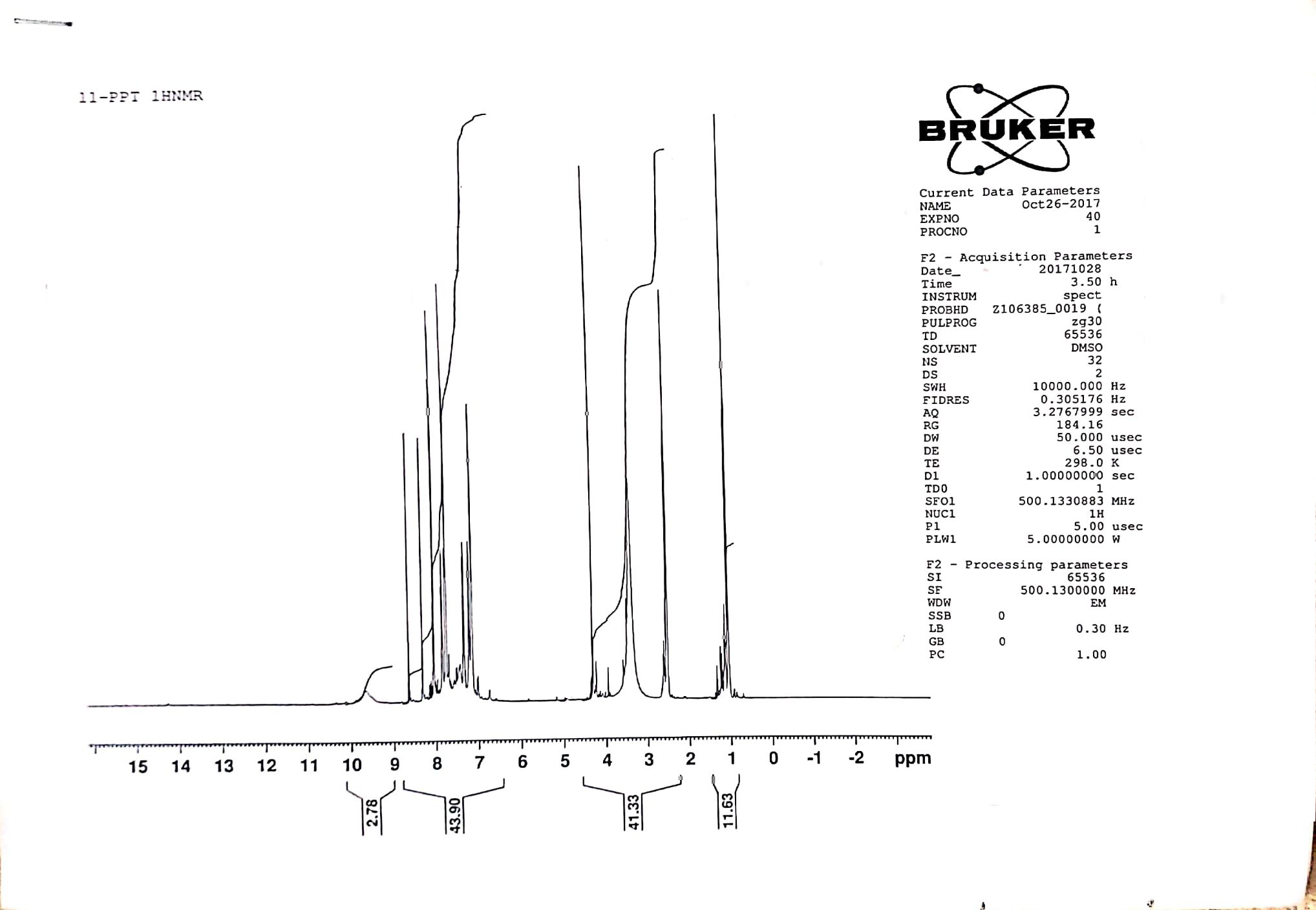


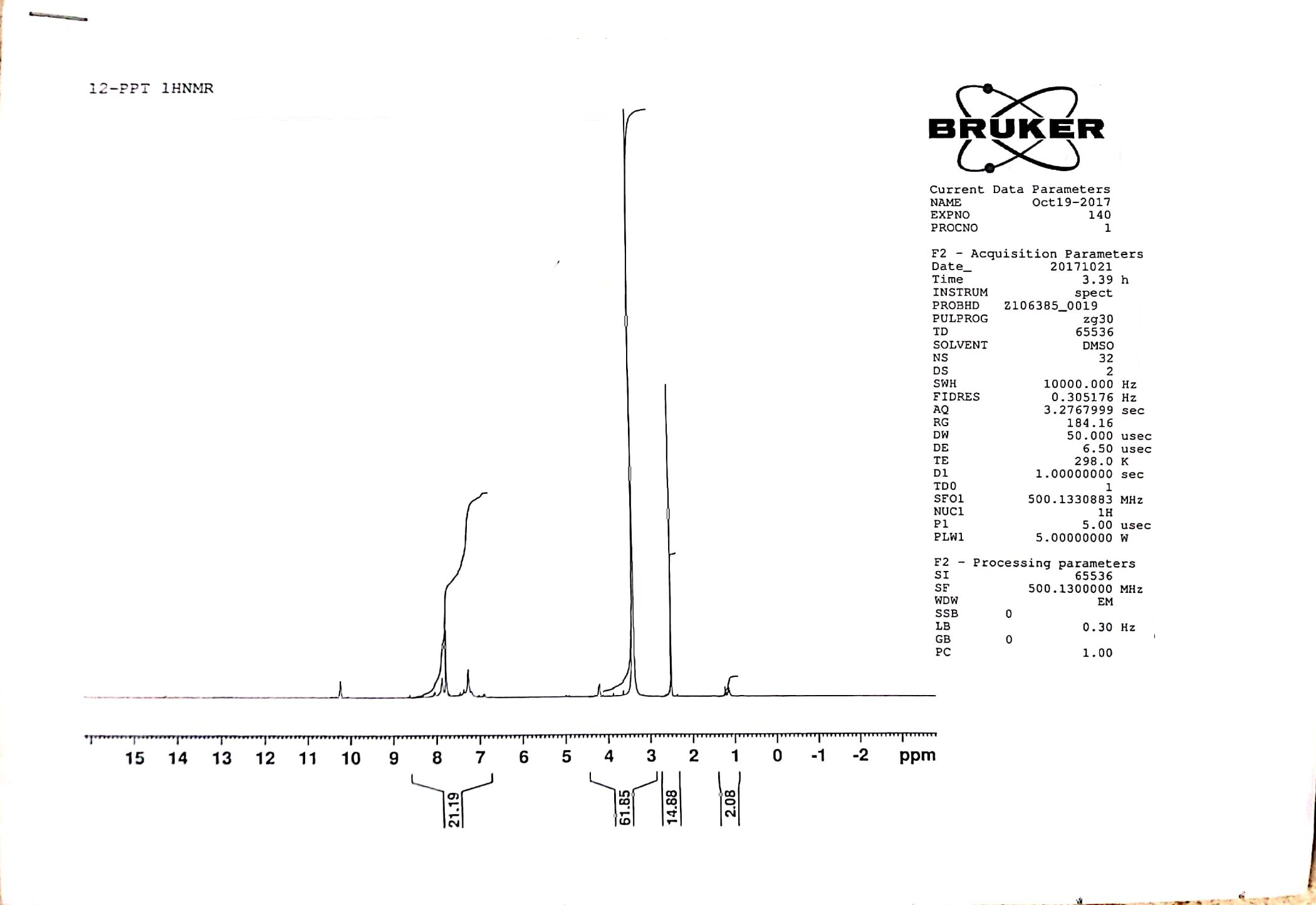


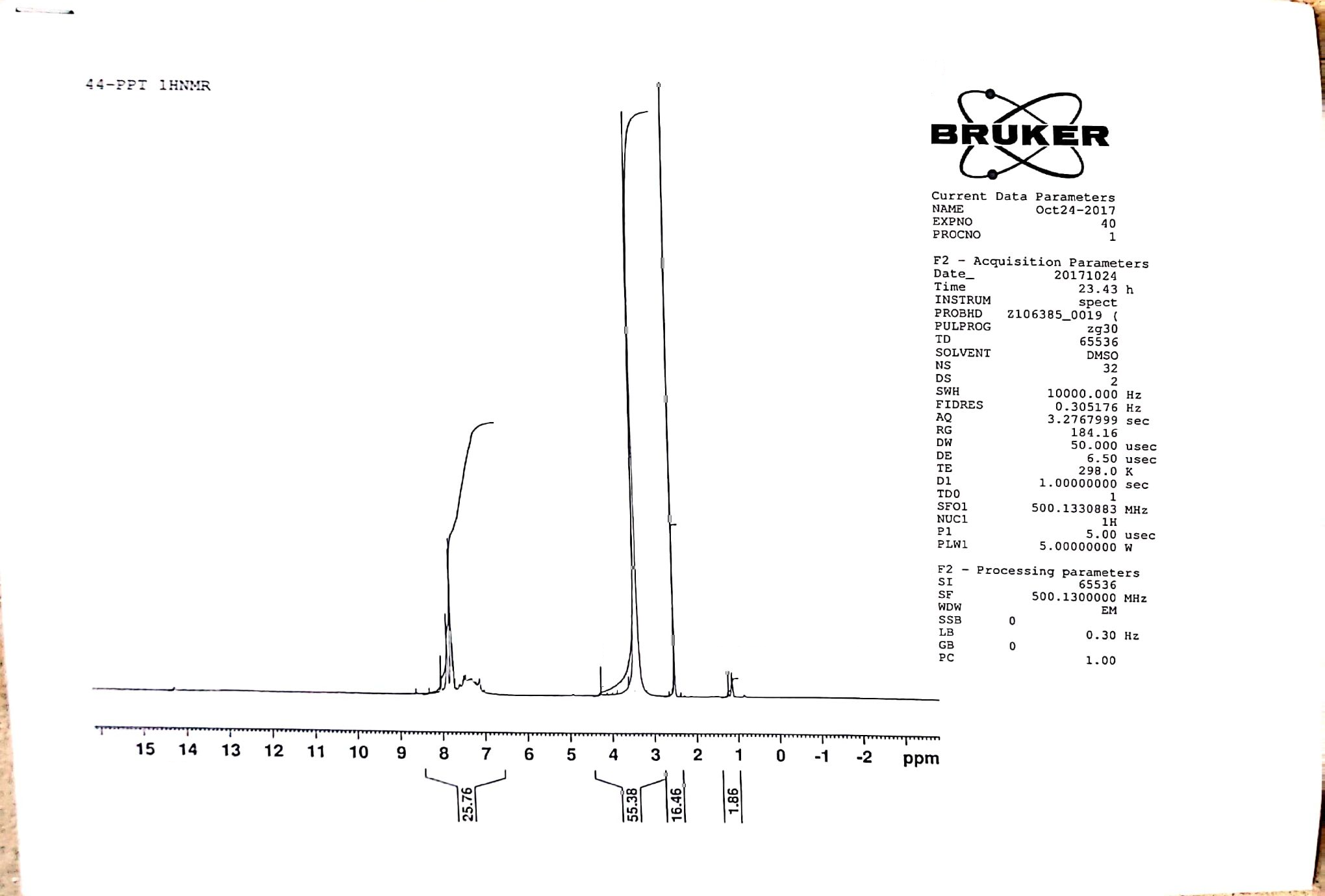


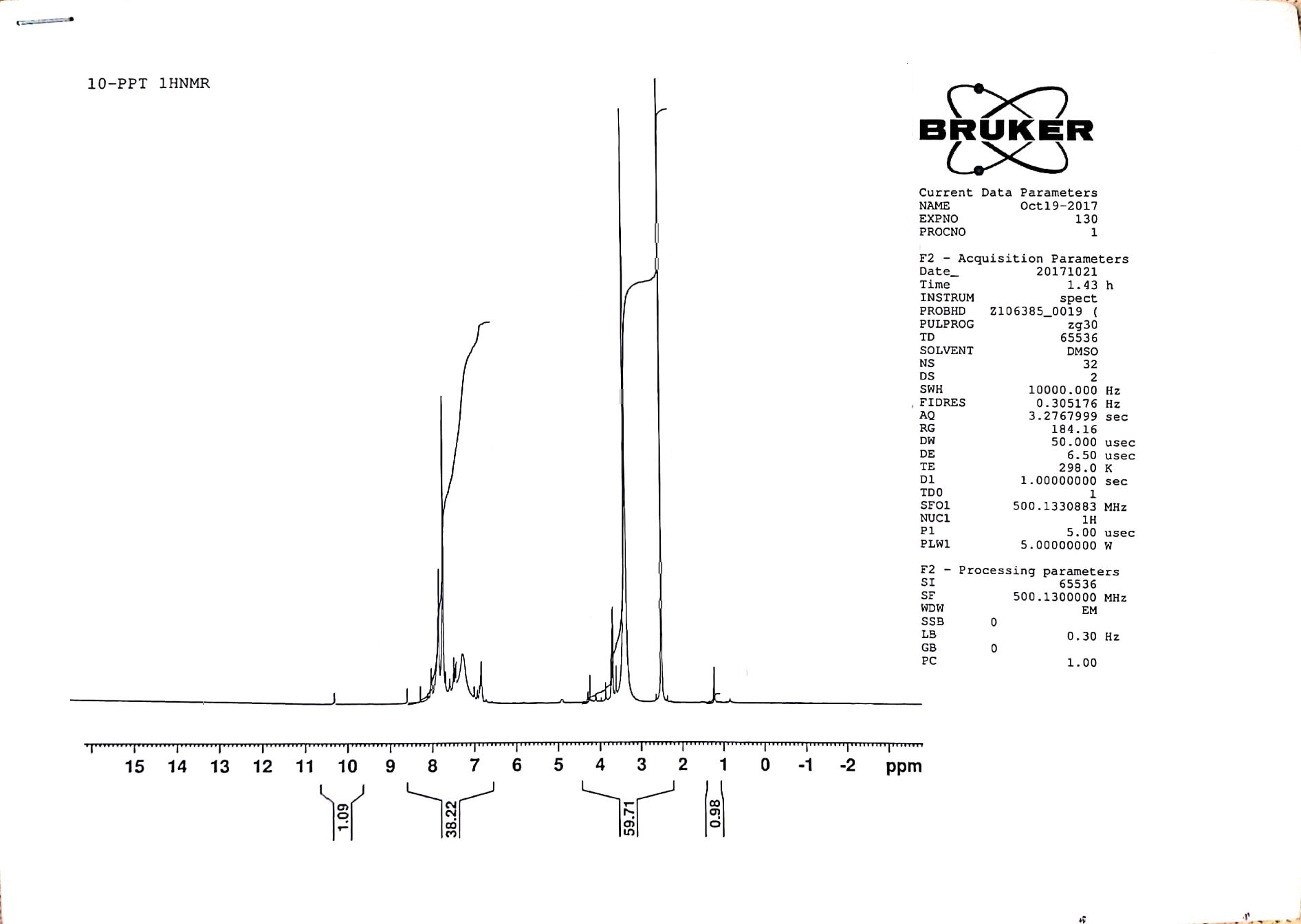


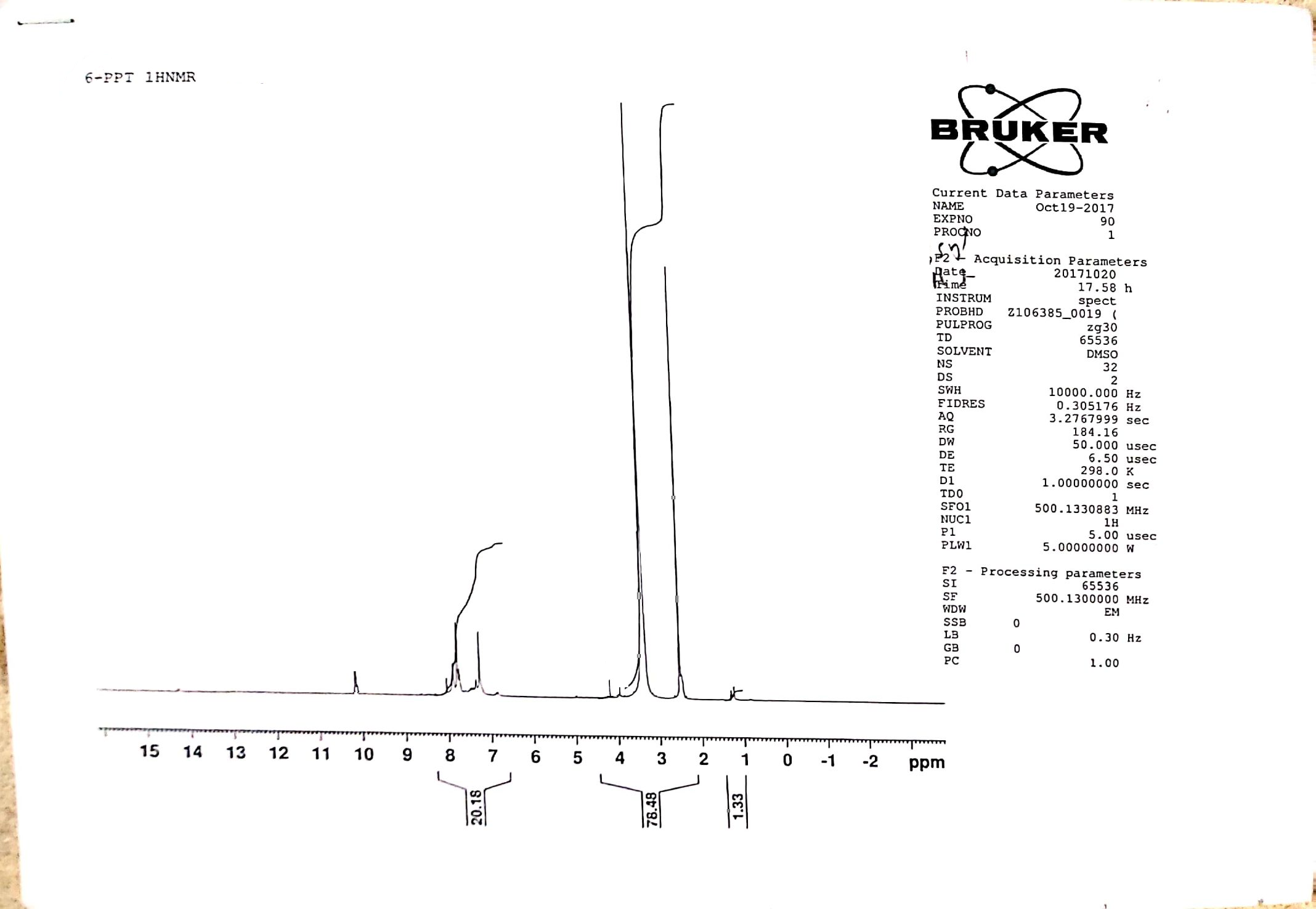


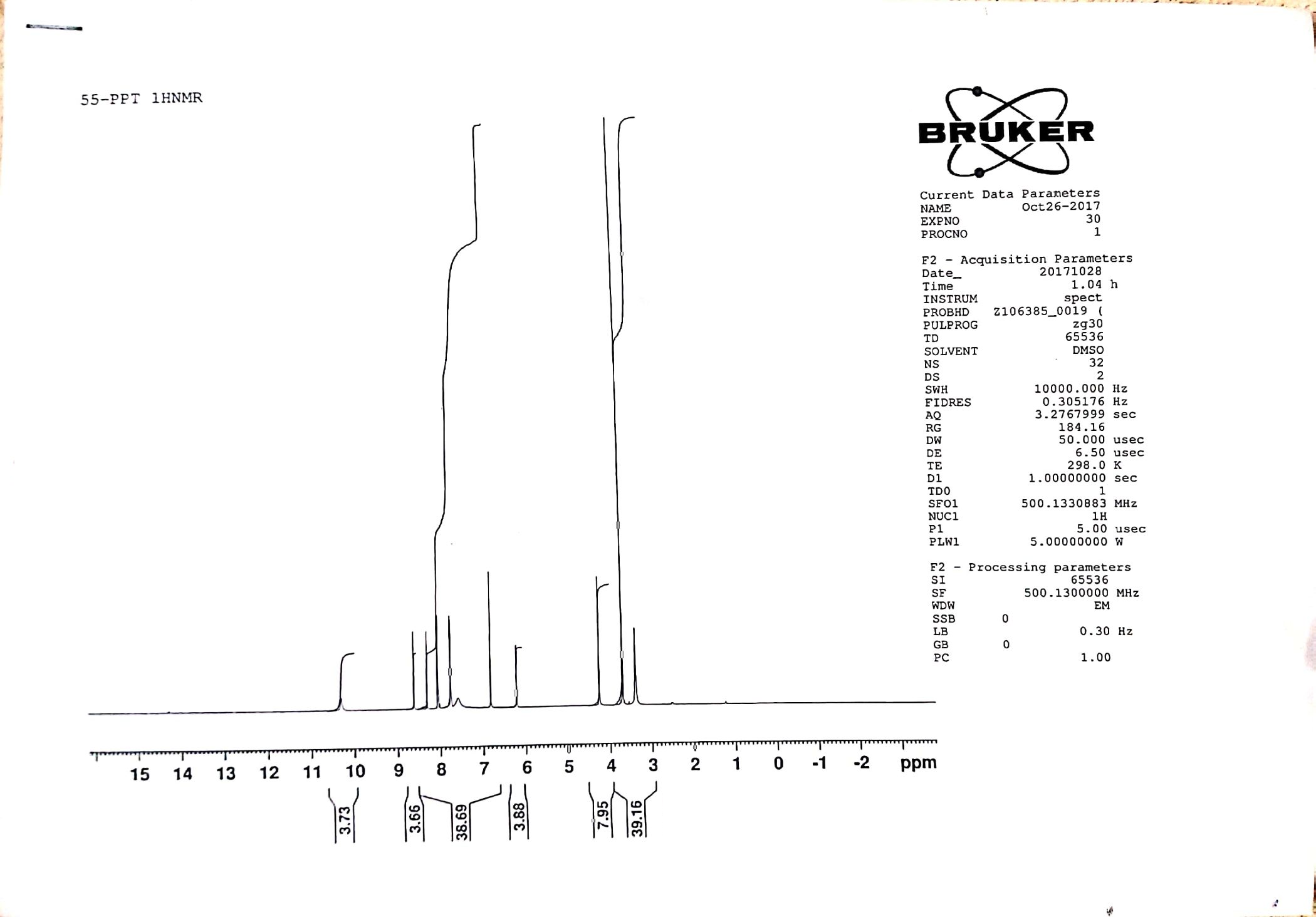


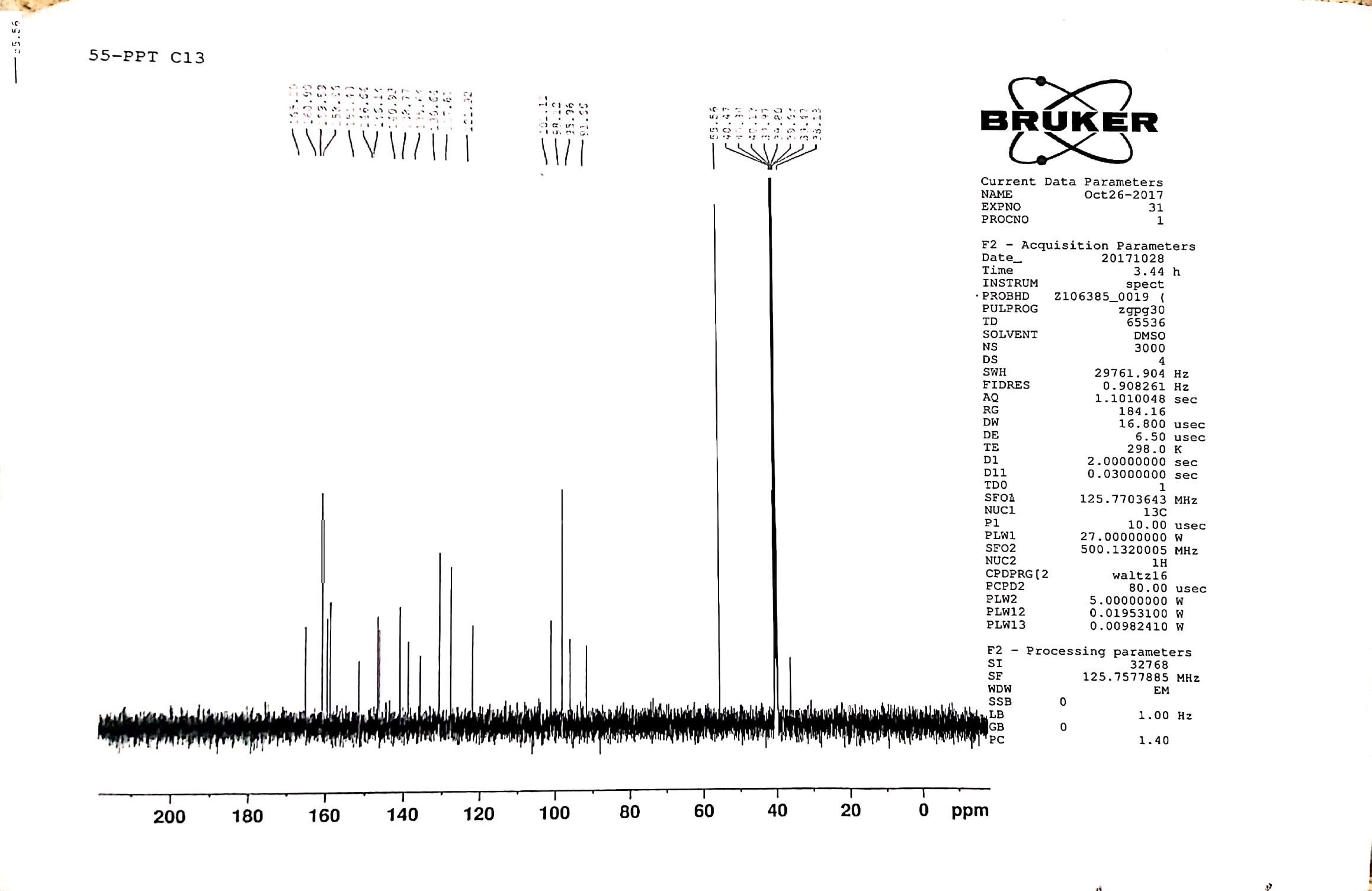


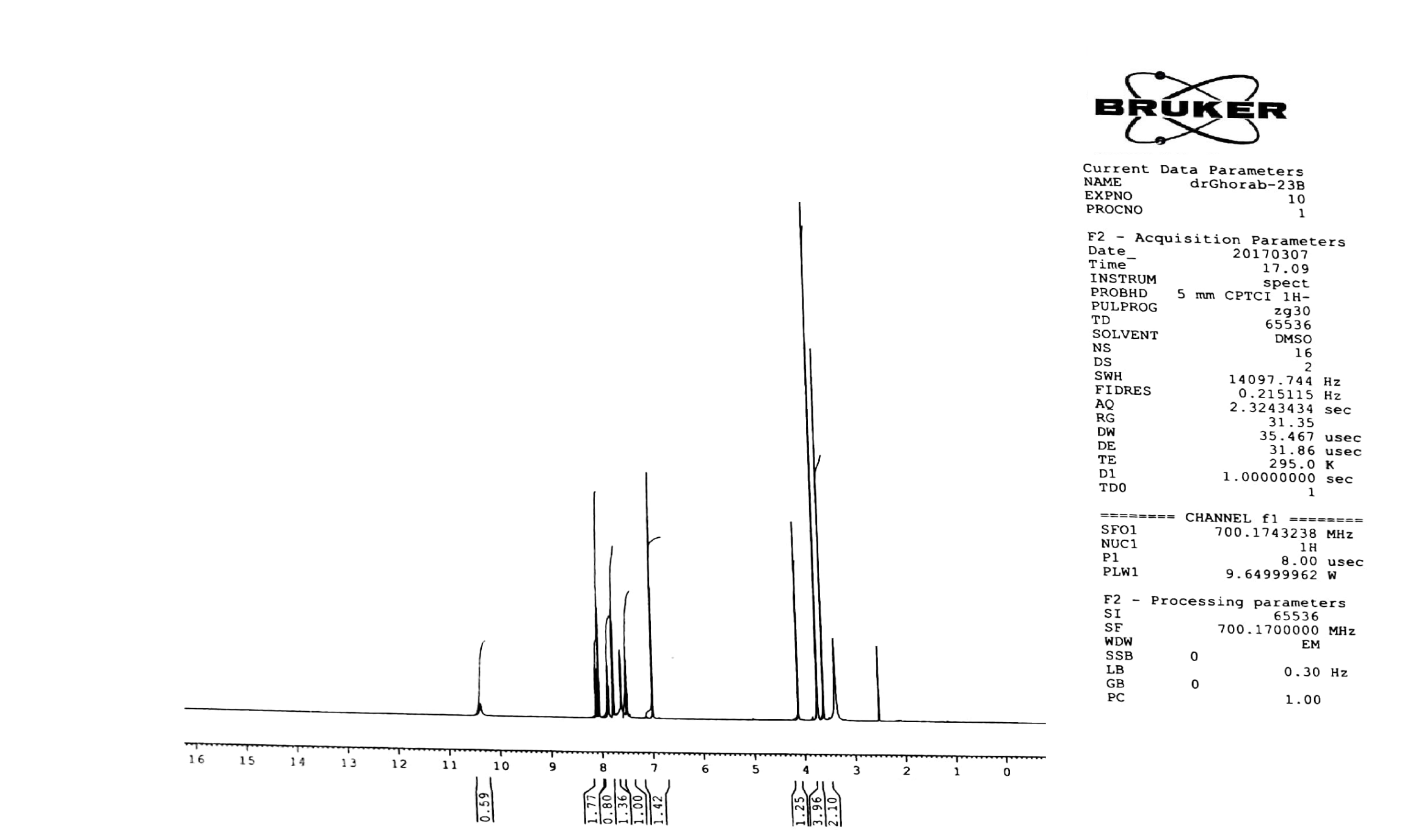


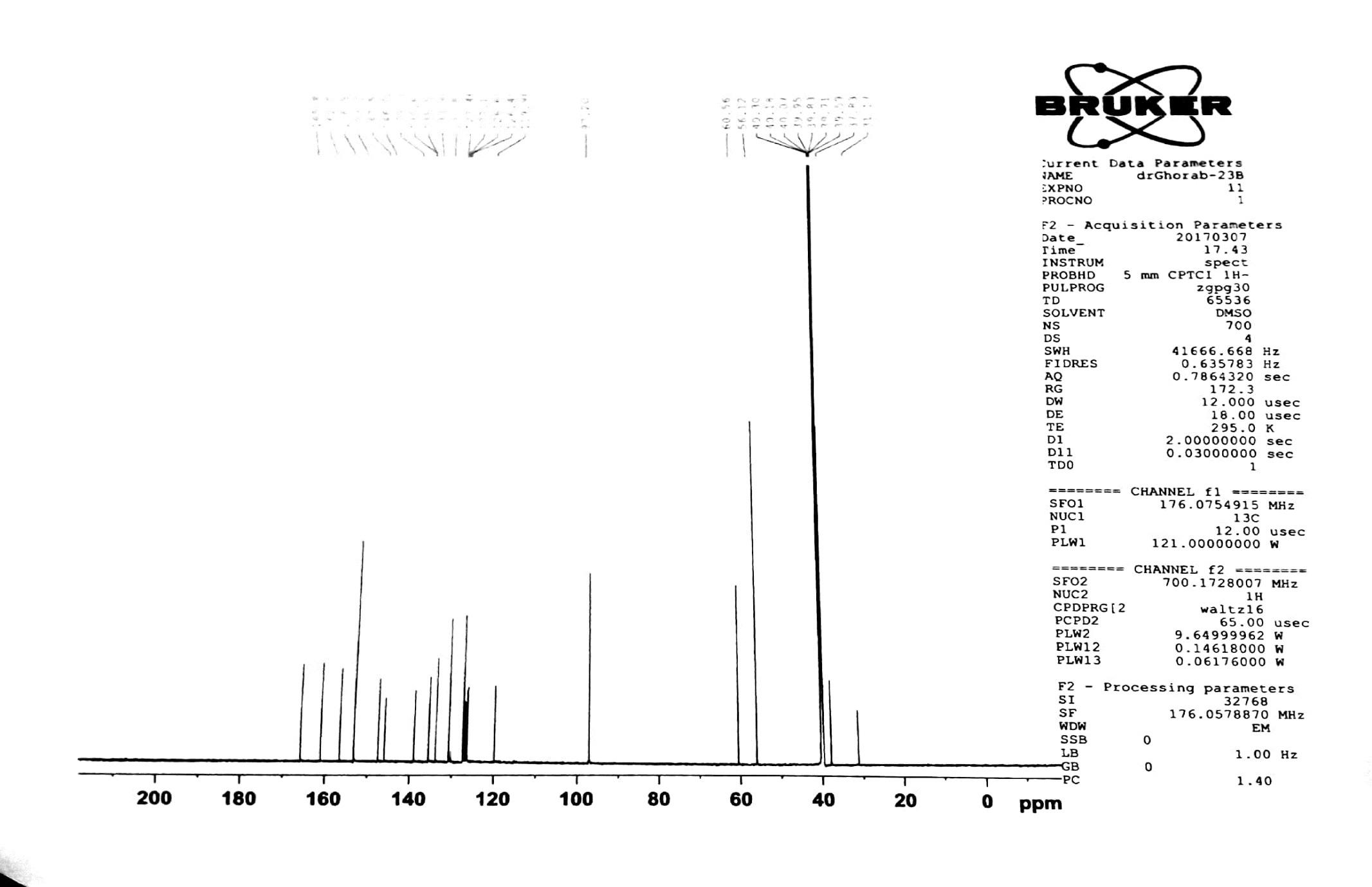


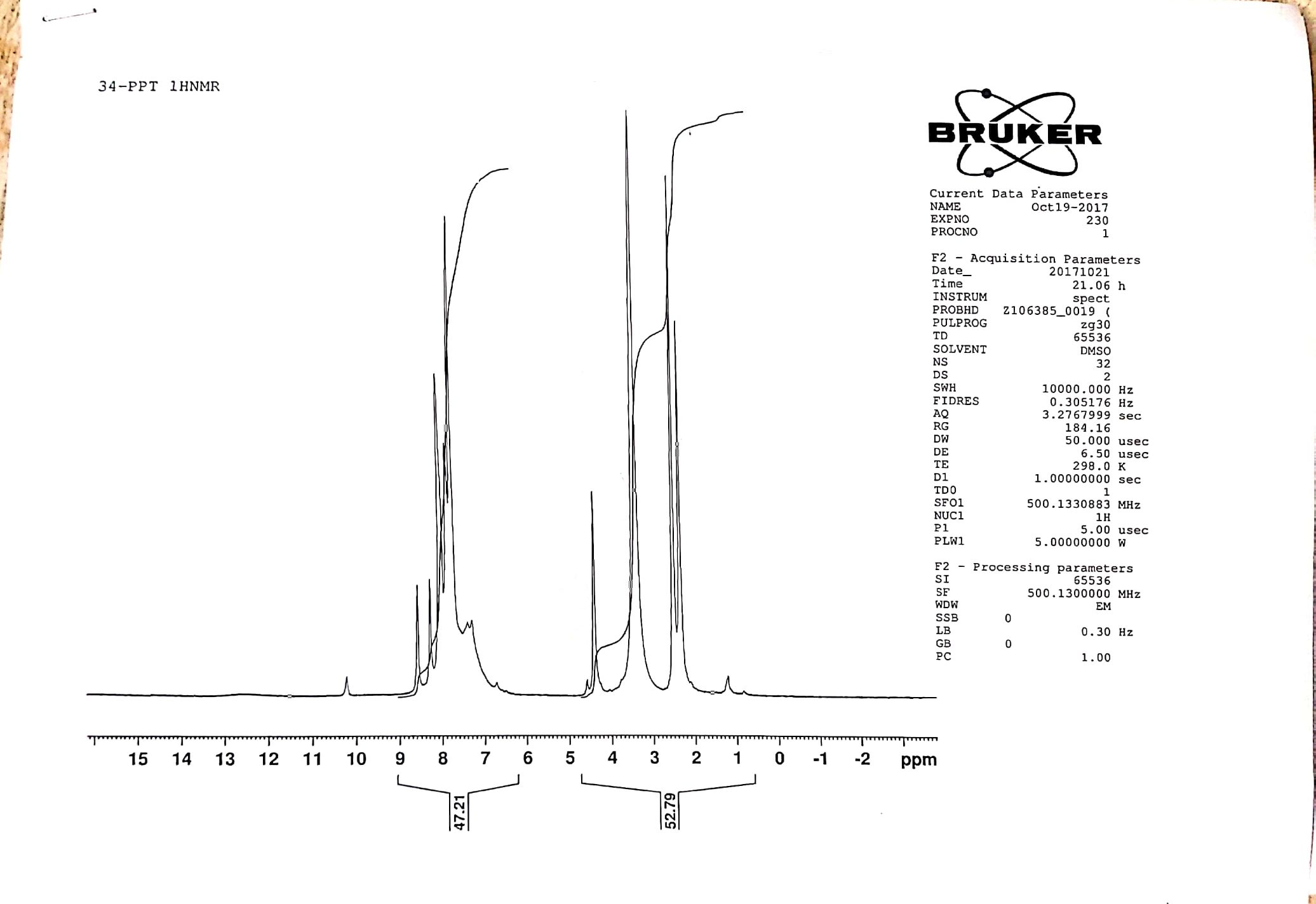


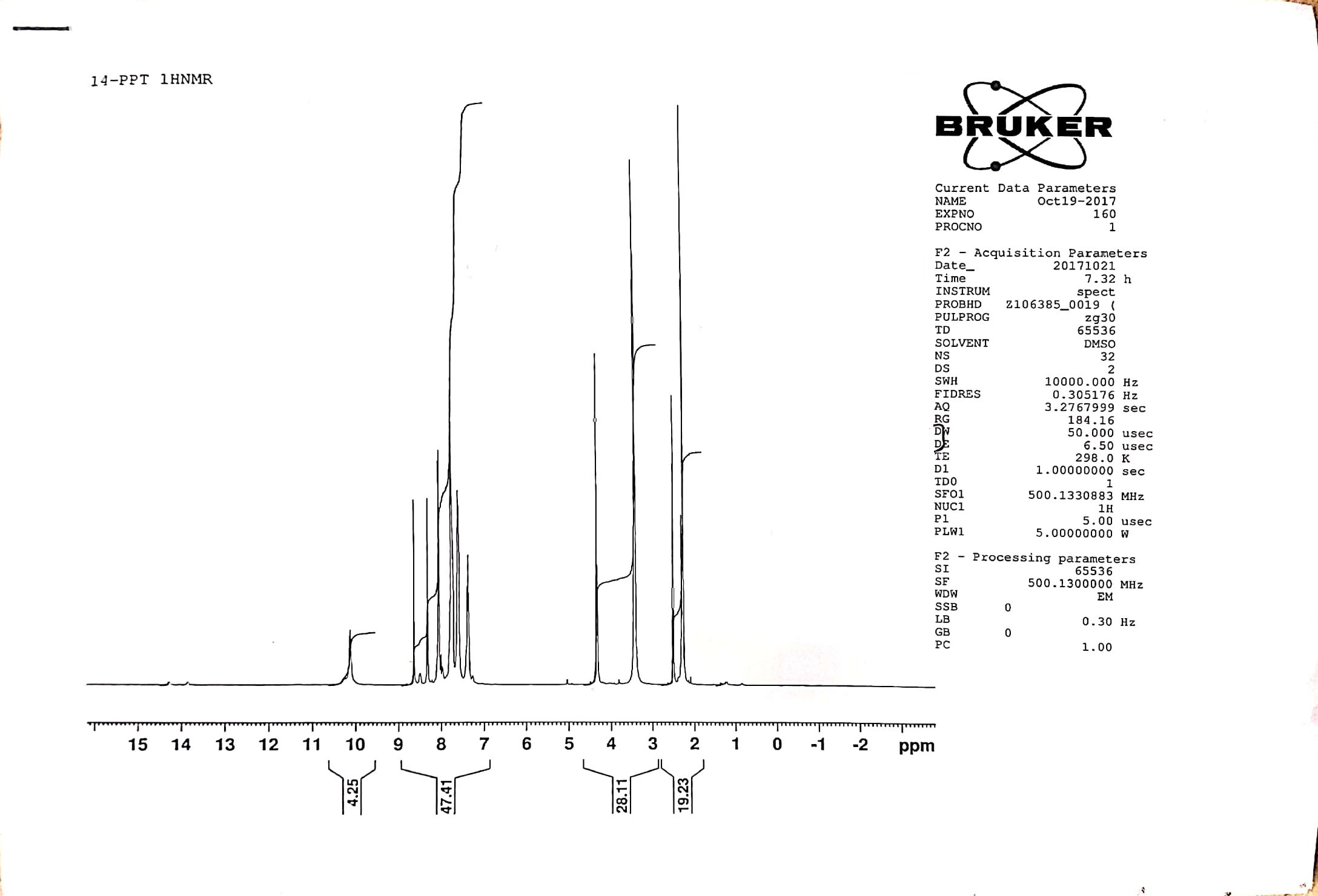


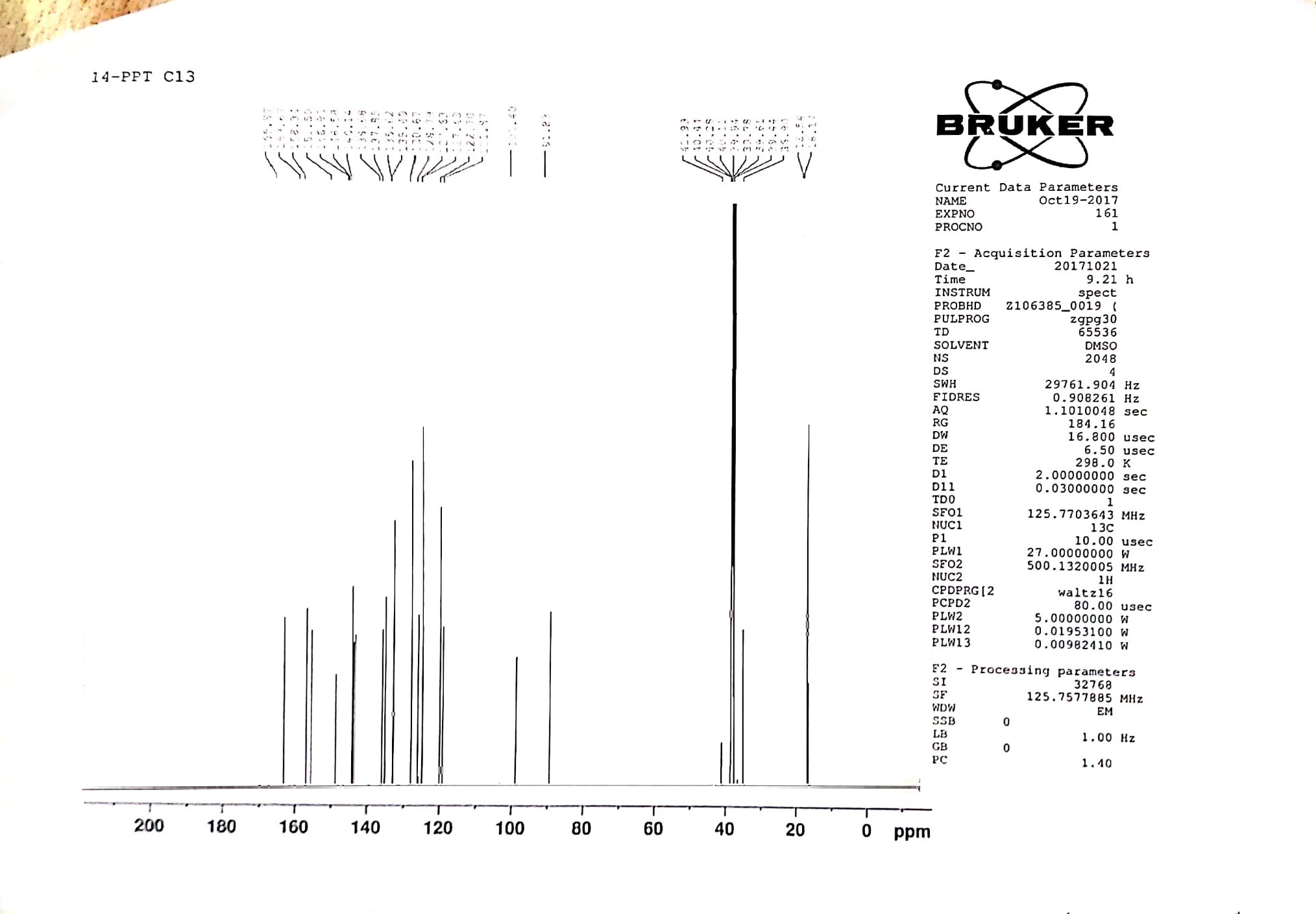


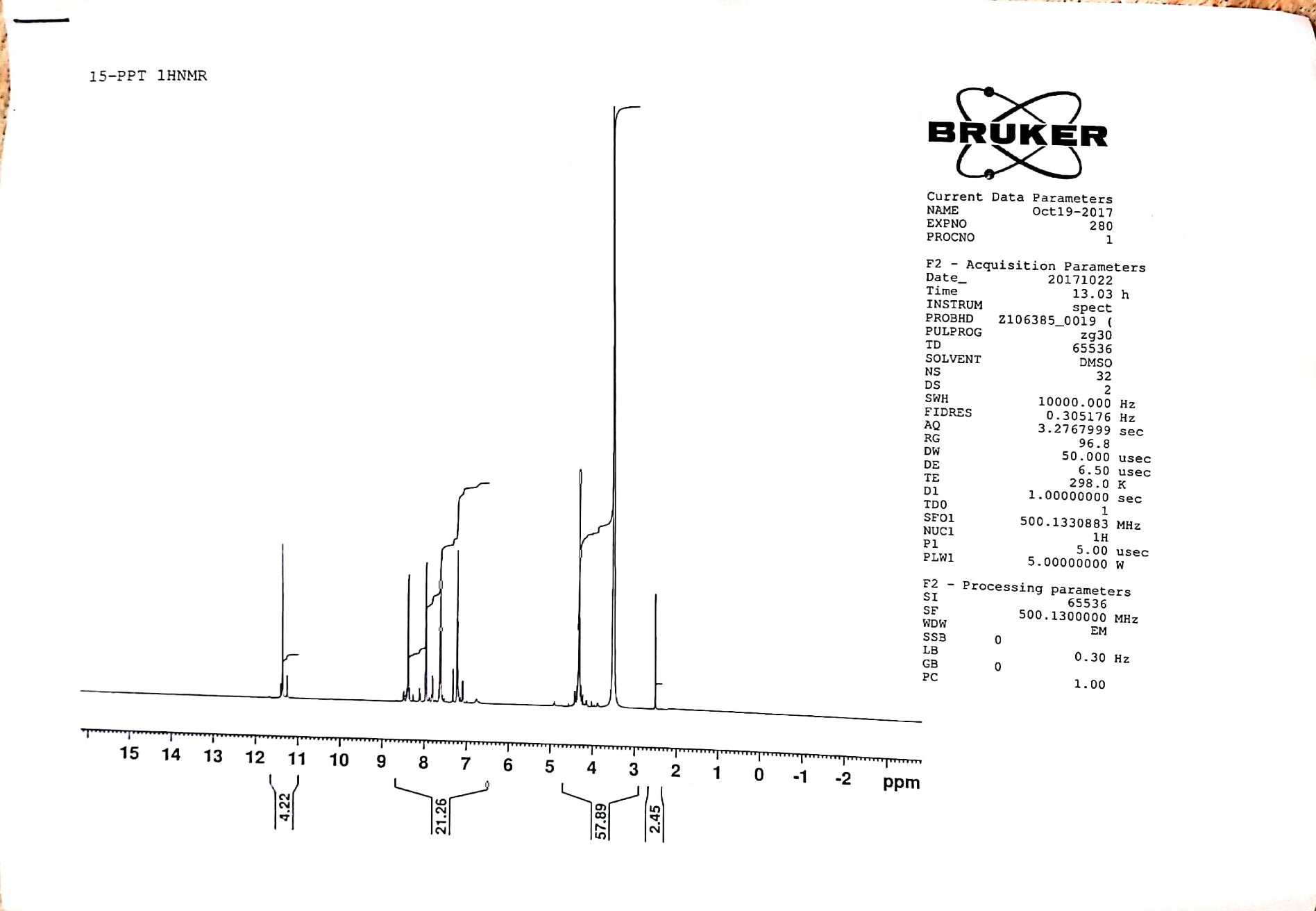

Supplement: Multimedia component 1 [file mmc1.docx]
